# Probabilistic Richardson extrapolation

**DOI:** 10.1093/jrsssb/qkae098

**Published:** 2024-12-26

**Authors:** Chris J Oates, Toni Karvonen, Aretha L Teckentrup, Marina Strocchi, Steven A Niederer

**Affiliations:** School of Mathematics, Statistics and Physics, Newcastle University, Newcastle upon Tyne, UK; School of Engineering Sciences, Lappeenranta–Lahti University of Technology LUT, Lappeenranta, Finland; Department of Mathematics and Statistics, University of Helsinki, Helsinki, Finland; School of Mathematics and Maxwell Institute for Mathematical Sciences, University of Edinburgh, Edinburgh, UK; National Heart and Lung Institute, Imperial College London, London, UK; School of Biomedical Engineering and Imaging Sciences, King’s College London, London, UK; National Heart and Lung Institute, Imperial College London, London, UK; School of Biomedical Engineering and Imaging Sciences, King’s College London, London, UK

**Keywords:** Bayesian statistics, Gaussian process, multi-fidelity modelling, reproducing kernel, uncertainty quantification

## Abstract

For over a century, extrapolation methods have provided a powerful tool to improve the convergence order of a numerical method. However, these tools are not well-suited to modern computer codes, where multiple continua are discretized and convergence orders are not easily analysed. To address this challenge, we present a probabilistic perspective on Richardson extrapolation, a point of view that unifies classical extrapolation methods with modern multi-fidelity modelling, and handles uncertain convergence orders by allowing these to be statistically estimated. The approach is developed using Gaussian processes, leading to *Gauss–Richardson Extrapolation*. Conditions are established under which extrapolation using the conditional mean achieves a polynomial (or even an exponential) speed-up compared to the original numerical method. Further, the probabilistic formulation unlocks the possibility of experimental design, casting the selection of fidelities as a continuous optimization problem, which can then be (approximately) solved. A case study involving a computational cardiac model demonstrates that practical gains in accuracy can be achieved using the GRE method.

## 1 Introduction

Testing of hypotheses underpins the scientific method, and increasingly these hypotheses are model-based. Deterministic or stochastic mathematical models are routinely used to represent mechanisms hypothesized to govern diverse phenomena, such as aerodynamics or electrochemical regulation of the human heart. In these cases, critical scientific enquiry demands a comparison of the model against a real-world dataset. The practical challenge is twofold; to simulate from the mathematical model, and to obtain a real-world dataset. Here, we focus on the first challenge—simulating from the model—which can be arbitrarily difficult depending on the complexity of the model. For example, simulating a single cycle of a jet engine to an acceptable numerical precision routinely requires $10^{6}$ core hours (Arroyo et al., 2021), while accurate simulation from the cardiac models that we consider later in this paper at steady state requires $10^{4}$ core hours in total (Strocchi et al., 2023). To drive progress in these and many other diverse scientific domains, there is an urgent need for statistical and computational methodology that can mitigate the high cost of accurately simulating from a mathematical model.

Abstractly, we enumerate all of the *discretization parameters* involved in approximate simulation from the mathematical model using scalars $x=(x_{1},\ldots ,x_{d})$, such that each component of x controls the error due to a particular aspect of discretization; for example, $x_{1}$ could be a time-step size, $x_{2}$ could be the width of a spatial mesh, and $x_{3}$ could be an error tolerance for an adaptive numerical method. The principal requirement is that the ideal mathematical model corresponds to the limit x→0, where no discretization is performed. Given a value of x, we denote as f(x) the associated numerical approximation to the continuum quantity f(0) from the mathematical model. The computational cost of such an evaluation will be denoted c(x), with c(0)=∞ being typical. The computational challenge addressed in this paper is to produce an accurate approximation to f(0), based on a dataset of simulations $\left\{f(x_{j})\right\}$, where $\{x_{j}\}\subset (0,\infty {)}^{d}$, such that the computational cost of obtaining $\left\{f(x_{j})\right\}$ remains within a prescribed budget. For this initial discussion, we focus on scalar-valued model output, but we generalize to multivariate and infinite-dimensional model output in Section 2.9.

Several solutions have been proposed to perform approximate simulation at a reduced cost. In what follows, it is useful to draw a distinction between *extrapolation methods*, applicable to the situation, where a mathematical model is discretized for simulation and numerical analysis of the discretization error can be performed, and *modern solutions* that are typically applied to ‘black box’ computer codes for which numerical analysis is impractical.

###  

####  

##### Extrapolation Methods

A unified presentation of extrapolation methods, that includes the most widely used algorithms, is provided by the so-called *E-algorithm* (see the survey of Brezinski, 1989). The starting point is a (real-valued) convergent sequence, which in our setting, we interpret as a sequence of numerical approximations $(f(x_{m}){)}_{m\in N}$, where $x_{m}$ is a vector of discretization parameters controlling the error in approximating the mathematical model, while f(0) represents the continuum quantity of interest. The E-algorithm posits an *ansatz* that


(1) $$f(x_{m})=f(0)+a_{1}g_{1}(m)+\ldots +a_{n-1}g_{n-1}(m)$$

for some unknown $a_{1},\ldots ,a_{n-1}\in R$, some known functions $g_{i}\;:\;N\to R$, and all m∈N. Then, instantiating (1) for m,m+1,…,m+n−1, we may solve for the unknown $a_{1},\ldots ,a_{n-1}$ and f(0) in terms of the *n* values $f(x_{m}),\ldots ,f(x_{m+n-1})$. Indeed, solving this linear system for f(0) leads to the estimator


(2) $$S_{m}\;:\;=S(f(x_{m}),\ldots ,f(x_{m+n-1}))=\frac{\left|\begin{array}{ccc} f(x_{m}) & \ldots & f(x_{m+n-1})\\ g_{1}(m) & \ldots & g_{1}(m+n-1)\\ \vdots & & \vdots \\ g_{n}(m) & \ldots & g_{n}(m+n-1) \end{array}\right|}{\left|\begin{array}{ccc} 1 & \ldots & 1\\ g_{1}(m) & \ldots & g_{1}(m+n-1)\\ \vdots & & \vdots \\ g_{n}(m) & \ldots & g_{n}(m+n-1) \end{array}\right|}.$$

Under appropriate assumptions, the sequence $(S_{m}{)}_{m\in N}$ constructed based on $(f(x_{m}){)}_{m\in N}$ as in (2) not only has the same limit, f(0), but also converges to that limit faster in the sense that ${\text{lim}}_{m\to \infty }(S_{m}-f(0))/(f(x_{m})-f(0))=0$; for precise statements, see Chapter 2 of Brezinski and Zaglia (2013).

The principal classes of extrapolation methods concern either the case of a single discretization parameter $x_{m}$, or they maintain ambivalence about $x_{m}$ by operating only on the values of the sequence $(f(x_{m}){)}_{m\in N}$. In either case, different extrapolation methods correspond to different basis functions $g_{i}$ in (1). *Richardson extrapolation* corresponds to $g_{i}(m)=x_{m}^{i}$, in which case (1) is recognized as polynomial extrapolation to the origin (Richardson, 1911; Richardson & Gaunt, 1927). The existence of a Taylor expansion of *f* at the origin is sufficient to guarantee a polynomial-rate convergence acceleration using Richardson’s method. Other examples of extrapolation methods include *Shanks’ transformation*  $g_{i}(m)=f(x_{m+i})-f(x_{m+i-1})$ (Shanks, 1955), the *Germain–Bonne transformation*  $g_{i}(m)=(f(x_{m+1})-f(x_{m}){)}^{i}$ (Germain-Bonne, 1990), and *Thiele’s rational extrapolation method*  $g_{i}(m)=x_{m}^{i}$, $g_{i+p}(m)=f(x_{m})x_{m}^{i}$ for i=1,…,p, n=2p+1 (Bulirsch & Stoer, 1964; Larkin, 1967; Thiele, 1909). A careful numerical analysis of *f* is usually required to determine when a particular extrapolation method can be applied. To the best of our knowledge, ideas from statistics and uncertainty quantification do not feature prominently, if at all, in the literature on extrapolation methods. In addition, the question of how best to construct the sequence $(x_{m}{)}_{m\in N}$ under a constraint on the overall computational budget does not appear to have been systematically addressed. Further background can be found in the book-level treatment of Sidi (2003) and Brezinski and Zaglia (2013).

Though rather classical, extrapolation methods continue to find new and useful applications, including in optimal transport (Chizat et al., 2020), regularization and training of machine learning models (Bach, 2021), and sampling with Markov chain Monte Carlo (Durmus et al., 2016).

##### Modern Solutions

If the mathematical model additionally involves one or more degrees of freedom θ, numerical approximations $f_{\theta }(x)$ are often required across a range of values for θ to identify configurations that are consistent with observations from the real world. Since the introduction of additional degrees of freedom further complicates numerical analysis, this setting has motivated the development of black box methods that can be applied in situations, where numerical analysis is impractical. Among these, *emulation* and *multi-fidelity modelling* (MFM) are arguably the most prominent.

In *emulation*, one attempts to approximate the map $\theta \mapsto f_{\theta }(x_{\mathrm{hi}-\mathrm{fi}})$, where the discretization parameters $x_{\mathrm{hi}-\mathrm{fi}}$ are typically fixed and correspond to a suitably high fidelity (hi-fi) model. This enables the prediction of computer code output at values of θ for which simulation was not performed (Sacks et al., 1989). A variety of sophisticated techniques have been developed to identify an appropriate basis or subspace in which an emulator can be constructed, such as *reduced order modelling* (Lucia et al., 2004). One drawback of emulation is that it can be *data hungry*; in applications for which it is only possible to perform a small number *n* of simulations, and for which insight from numerical analysis is unavailable, one usually cannot expect to obtain high-quality predictions. A second drawback is that emulation treats the discretized model $\theta \mapsto f_{\theta }(x_{\mathrm{hi}-\mathrm{fi}})$ as the target, whereas in reality, the continuum mathematical model $\theta \mapsto f_{\theta }(0)$ is of principal interest.

A partial solution to the drawbacks of emulation is MFM, in which one supplements a small number of simulations from the hi-fi model $\theta \mapsto f_{\theta }(x_{\mathrm{hi}-\mathrm{fi}})$ with a larger number of simulations from one or more cheaper low fidelity (lo-fi) models $\theta \mapsto f_{\theta }(x_{\mathrm{lo}-\mathrm{fi}})$ (Peherstorfer et al., 2018). Lo-fi models can sometimes be obtained using coarse-grid approximations, early stopping of iterative algorithms, or linearization (Piperni et al., 2013). Alternatively, lo-fi models could involve only a subset of the relevant physical mechanisms, an approach popular, e.g. in climate science (Held, 2005; Majda & Gershgorin, 2010). Once specified, the models of different fidelities can be combined in different ways: one can either use the hi-fi model to periodically ‘check’ (and possibly adapt) the lo-fi models; or one can use the lo-fi models as pilot runs to decide whether or not to evaluate the hi-fi model; or one can use the information from all models simultaneously, by defining a multi-fidelity surrogate model (Craig et al., 1998; Cumming & Goldstein, 2009; Ehara & Guillas, 2023; Kennedy & O’Hagan, 2000), where correlation between models is taken into account. Provided that the lo-fi models are correlated with the original model, these additional cheap simulations can be leveraged to more accurately predict computer code output. The principal drawback of MFM is that there is limited guidance on how the lo-fi models should be constructed, and a poor choice can fail to improve (or even worsen) predictive performance, while incurring an additional computational cost. In addition, as with emulation, the literature on MFM tends to treat the hi-fi model as the target, rather than the continuum mathematical model.

##### Other Related Work

Some alternative lines of research will briefly be discussed. *Probabilistic numerics* casts numerical approximation as a statistical task (Hennig et al., 2015), with Bayesian principles used to quantify uncertainty regarding the continuum model of interest (Cockayne et al., 2019). However, the focus of the literature is the design of numerical methods, in contrast to extrapolation methods, which operate on the output of existing numerical methods. In parallel, the application of machine learning methods to numerical tasks has received recent attention; for example, deep learning is being used for numerical approximation of high-dimensional parametric partial differential equations (Han et al., 2018). This literature does not attempt extrapolation as such, with a hi-fi numerical method typically used to provide a training dataset. Gaussian processes have been used in specific applications to extrapolate a series of numerical approximations to a continuum quantity of interest f(0), for example, in Thodoroff et al. (2023) to model ice sheets in Antarctic, and in Ji et al. (2024) to model the evolution of the quark-gluon plasma following the Big Bang. To date, however, convergence acceleration has not been studied in the Gaussian process context. An important numerical task encountered in statistics is to approximate an expected value of interest f(0)=E[X(0)]. Unbiased estimation of f(0) at finite cost is possible in this setting using the methodology of Rhee and Glynn (2015), provided one can construct a sequence $(X(x_{n}){)}_{n\in N}$ of computable stochastic approximations to X(0), such that the variance of $X(x_{n})-X(x_{n-1})$ decays sufficiently fast. Similar de-biasing ideas have since been used in the context of Markov chain Monte Carlo (Jacob et al., 2020). Multilevel methods, based on such sequences, have been combined with Richardson extrapolation in Lemaire and Pagès (2017) and Beschle and Barth (2022).

##### Our Contribution

This paper proposes a probabilistic perspective on extrapolation methods that unifies extrapolation methods and multi-fidelity modelling (MFM). The approach is instantiated using a *numerical analysis-informed Gaussian process* to approximate the map x↦f(x), as described in Section 2, where the conditional mean can be interpreted as a (novel) extrapolation method, in the sense that it provably achieves a polynomial (or even an exponential) speed-up compared to the original numerical method. Like Richardson extrapolation, our theoretical arguments are rooted in Taylor expansions, so the name Gauss–Richardson Extrapolation (GRE) is adopted. The probabilistic formulation of extrapolation methods confers several advantages:

- In contrast to classical extrapolation methods, which focus on the case of a univariate discretization parameter $x_{n}$, it is straight-forward to consider a vector of discretization parameters $x_{n}$ within a regression framework. In Sections 2.1–2.3, the probabilistic approach is laid out, then in Sections 2.4 and 2.5, higher-order convergence guarantees for GRE are established.
- Credible sets for the continuum quantity of interest f(0) can be constructed, enabling computational uncertainty to be integrated into experimental design and downstream decision-support. The asymptotic performance of GRE credible sets is analysed in Section 2.6.
- In contrast to existing approaches in MFM, where a discrete set of fidelities are specified at the outset, GRE operates on a continuous spectrum of fidelities and casts the selection of fidelities as a cost-constrained experimental design problem, which can then be approximately solved using methods described in Section 2.7.
- For computer models whose convergence order is difficult to analyse, the probabilistic formulation allows for convergence orders to be formally estimated. The consistency of a maximum quasi-likelihood approach to estimating unknown convergence order is established in Section 2.8.

The methodology is rigorously tested in the context of simulating from a computational cardiac model involving separate spatial and temporal discretization parameters in Section 3. The sensitivity of the cardiac model to the different discretization parameters is first estimated from lo-fi simulations, then an optimal experimental design is generated and used to estimate the true trajectory of the cardiac model in the continuum limit. Our experimental results demonstrate that a practical gain in accuracy can be achieved with our GRE method. Though our assessment focuses on a specific cardiac model of scientific and clinical interest, the methodology is general and offers an exciting possibility to accelerate computation in the diverse range of scenarios in which computationally intensive simulation is performed. A closing discussion is contained in Section 4.

Code to reproduce our results is provided at https://github.com/christopheroates/Richardson.

## 2 Methodology

This section contributes a probabilistic perspective on extrapolation methods, which we instantiate using Gaussian processes (GPs) to produce Gauss–Richardson Extrapolation (GRE). For simplicity of presentation, we first consider the case of a scalar quantity of interest, generalizing to arbitrary-dimensional quantities of interest in Section 2.9.

###  

####  

##### Set-Up

Let f:X→R be a (nonrandom) real-valued function on a bounded set $X\subset [0,\infty {)}^{d}$ such that 0∈X. As in Section 1, the output f(x) for x≠0 will represent a numerical approximation to a continuum quantity f(0) of interest, and for extrapolation to be possible at all we will minimally need to assume that *f* is continuous at 0.

##### Notation

For $\beta \in N_{0}^{p}$, let $\partial^{\beta }g$ denote the mixed partial derivative $x\mapsto \partial_{x_{1}}^{\beta_{1}}\ldots \partial_{x_{p}}^{\beta_{p}}\;g(x)$ of a function g:D→R, whenever this is well-defined and $D\subseteq R^{p}$. Let $C^{s}(D)$ denote the set of *s*-times continuously differentiable functions g:D→R, meaning that $\partial^{\beta }g$ is continuous for all |β|≤s, where $|\beta |\;:\;=\beta_{1}+\cdots +\beta_{p}$. For g:D→R bounded, let $\left\|g{\|}_{L^{\infty }(D)}\;:\;={\text{sup}}_{x\in D}|g(x)\right|$. Let $\pi_{r}(D)$ denote the set of all polynomial functions of total degree at most *r* on *D*. For vectors $a,b\in R^{d}$, let $[a,b]\;:\;=[a_{1},b_{1}]\times \ldots \times [a_{d},b_{d}]\subset R^{d}$, and similarly for [a,b) and so forth. Let GP(m,k) denote the law of a GP with mean function *m* and covariance function *k*; background on GPs can be found in Rasmussen and Williams (2006).

### 2.1 A numerical analysis-informed Gaussian process

**Figure 1.**
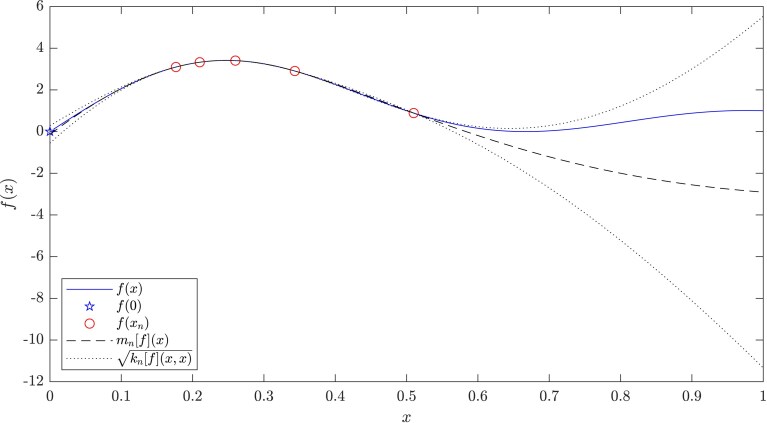
The numerical analysis-informed Gaussian process model, fitted to an illustrative dataset $\{f(x_{i}){\}}_{i=1}^{n}$ (circles) of size n=5, corresponding to the approximations produced by a finite difference method (solid curve) whose first-order accuracy [i.e. b(x)=x] was encoded into the GP. The scale $\sigma_{n}^{2}[f]$ of the uncertainty was calibrated using the method advocated in Section 2.6, while $k_{e}$ was taken to be a Matérn-$\frac{5}{2}$ kernel with length-scale parameter selected using quasi-maximum likelihood (see Section 2.8). Observe that point estimate $m_{n}[f](0)$ (dashed curve at x=0), is more accurate than that of the highest fidelity simulation from the numerical method, while the limiting quantity of interest f(0) (star) falls within the one standard deviation prediction interval (dotted curves at x=0).

Assuming for the moment that numerical analysis of x↦f(x) can be performed, our first aim is to encode the resulting bounds on discretization error into a statistical regression model. Training such a *numerical analysis-informed* regression model on data $\{f(x_{i}){\}}_{i=1}^{n}$ obtained at distinct inputs $X_{n}=\{x_{i}{\}}_{i=1}^{n}\subset X\setminus \{0\}$ enables statistical prediction of the limit f(0), in analogy with classical extrapolation methods. To leverage conjugate computation, here we instantiate the idea using GPs in a Bayesian framework. For the first part, we require an explicit error bound b:X→[0,∞) such that b(x)≥0 with equality if and only if x=0, and such that f(x)−f(0)=O(b(x)). The error bound *b* will be encoded into a centred prior GP model for *f*, whose covariance function


(3) $$k(x,x^{\prime })\;:\;=\sigma^{2}\left[k_{0}^{2}+b(x)b(x^{\prime })k_{e}(x,x^{\prime })\right],\;x,x^{\prime }\in X,$$

is selected to ensure samples g∼GP(0,k) from the GP satisfy, with probability one, g(x)−g(0)=O(b(x)) (see Appendix B.1 for the precise statement). Here, $\sigma^{2}>0$ is an overall scale to be estimated, while the scalar $k_{0}^{2}>0$ is proportional to the prior variance of f(0). The symmetric positive-definite function $k_{e}\;:\;X\times X\to R$ is the covariance function for the *normalized error*  x↦e(x), where $e(x)\;:\;=b(x{)}^{-1}(f(x)-f(0))$ for x∈X∖{0}, and must be specified. In practice, $k_{e}$ will additionally involve length-scale parameters ℓ which must be estimated, for example $k_{e}(x,x^{\prime })=\text{exp}(-\sum_{i=1}^{d}\ell_{i}^{-2}(x_{i}-x_{i}^{\prime }{)}^{2})$ in the case of the Gaussian kernel; we defer all discussion of this point to Sections 2.8 and 3. To our knowledge, the encoding of convergence orders into a GP as in (3) has not been well-studied, though the basic idea appeared in Tuo et al. (2014) and Bect et al. (2021) and in our preliminary work (Teymur et al., 2021). Standard techniques can be applied to fit such a GP model to a dataset; see Figure 1 and Section 2.2.

Remark 1 **Recovering Richardson in dimension d=1**

Let $k_{e}$ be any kernel that reproduces the polynomial space $\pi_{n-2}(R)$, such as $k_{e}(x,x^{\prime })=(1+xx^{\prime }{)}^{n-2}$, and consider the ‘objective’ prior with $k_{0}^{2}\to \infty$. Conditioning on data $\{f(x_{i}){\}}_{i=1}^{n}$, the posterior mean function is the unique interpolant of the form x↦μ+b(x)p(x) for some μ∈R, $p\in \pi_{n-2}(R)$ (see e.g. Karvonen et al., 2018, Proposition 2.6). Thus, if *b* is polynomial, the intercept *μ* is the result of polynomial extrapolation to 0, and is an instance of Richardson’s classical extrapolation method.

Unfortunately the connection in Remark 1 is not especially useful. Indeed, while the posterior mean provides a useful point estimate, the posterior variance is identically zero, meaning that predictive uncertainty is not being properly quantified. Thus, we do not attempt to reproduce Richardson extrapolation in the sequel, but rather, we develop *de novo* methodology tailored to the GP framework.

### 2.2 Gauss–Richardson extrapolation

First we recall the relevant calculations for conditioning the GP model (3) on a dataset. Let $k_{b}\;:\;X\times X\to R$ be defined as $k_{b}(x,x^{\prime })\;:\;=b(x)b(x^{\prime })k_{e}(x,x)$, so that our assumptions on *b* and $k_{e}$ imply that $k_{b}$ is a symmetric positive-definite kernel on X∖{0}, and a symmetric positive semi-definite kernel on X. Let $f(X_{n})$ be a column vector with entries $f(x_{i})$, let $k_{b}(x)$ be a column vector with entries $k_{b}(x_{i},x)$, and let $K_{b}$ be a matrix with entries $k_{b}(x_{i},x_{j})$. Recalling that $k_{0}^{2}$ is proportional to the prior variance for f(0), we opt for an ‘objective’ prior in which $k_{0}^{2}\to \infty$. However, this limit results in an improper prior GP. To make progress, we must first compute the conditional GP using a finite value of $k_{0}^{2}$ and then retrospectively take the limit—a standard calculation which we detail in Appendix B.2—yielding conditional mean and covariance functions


(4) $$m_{n}[f](x)\;:\;=\frac{1^{⊤}K_{b}^{-1}f(X_{n})}{1^{⊤}K_{b}^{-1}1}+k_{b}(x{)}^{⊤}K_{b}^{-1}\left\{\;f(X_{n})-\left(\frac{1^{⊤}K_{b}^{-1}f(X_{n})}{1^{⊤}K_{b}^{-1}1}\right)1\right\},$$

(5) $$k_{n}[f](x,x^{\prime })\;:\;=\sigma_{n}^{2}[f]\left\{k_{b}(x,x^{\prime })-k_{b}(x{)}^{⊤}K_{b}^{-1}k_{b}(x^{\prime })+\frac{\left[k_{b}(x{)}^{⊤}K_{b}^{-1}1-1][k_{b}(x^{\prime }{)}^{⊤}K_{b}^{-1}1-1\right]}{1^{⊤}K_{b}^{-1}1}\right\},$$

where 1 is a column vector whose elements are all 1. The matrix $K_{b}$ can indeed be inverted since we have assumed that the entries of $X_{n}\subset X\setminus \{0\}$ are distinct. To obtain (5), we have additionally replaced $\sigma^{2}$ with $\sigma_{n}^{2}[f]$, an estimator for the scale parameter *σ*, to be specified in Section 2.6. Computing the conditional mean and variance at x=0 results in the simple formulae


(6) $$m_{n}[f](0)=\frac{1^{⊤}K_{b}^{-1}f(X_{n})}{1^{⊤}K_{b}^{-1}1}\;\text{and}\;k_{n}[f](0,0)=\frac{\sigma_{n}^{2}[f]}{1^{⊤}K_{b}^{-1}1},$$

since b(0)=0, and thus $k_{b}(0,x)=b(0)b(x)k_{e}(0,x)=0$ for all x∈X. The proposed GRE method returns a (univariate) Gaussian distribution, which can be summarized using the point estimate $m_{n}[f](0)$ for f(0), together with the 100(1−α)% credible intervals


(7) $$C_{\alpha }[f]=\left\{y\in R\;:\;\frac{\left|y-m_{n}[f](0)\right|}{\sqrt{k_{n}[f](0,0)}}\leq \Phi^{-1}\left(1-\frac{\alpha }{2}\right)\right\},$$

where Φ denotes the standard Gaussian cumulative density function. The uncertainty quantification provided by GRE unlocks additional functionality that was not available to classical extrapolation methods, including optimal experimental design for selecting $X_{n}$ (Section 2.7) and principled statistical methods for estimating uncertain convergence orders (Section 2.8). However, both the accuracy of the point estimate and the coverage of the credible intervals will depend critically on the choice of the scale estimator $\sigma_{n}^{2}[f]$ and the choice of covariance function $k_{e}$. This important issue of how to select $\sigma_{n}^{2}[f]$ and $k_{e}$ will be discussed next. An illustration of the proposed GRE method is provided in Figure 1.

### 2.3 Conservative Gaussian process priors

Our set-up involves a nonrandom function *f* that is modelled using a prior GP. One would perhaps hope to elicit a prior covariance function *k* in such a manner that *f* could plausibly have been generated as a sample from the GP. However, such elicitation is fundamentally difficult; the sample support set of a GP is not a vector space and may not even be measurable in general (Karvonen, 2023; Stein & Hung, 2019). How then can we proceed? In the applications that we have in mind, it is often possible to identify a symmetric positive-definite kernel such that *f* belongs to the reproducing kernel Hilbert space (RKHS) associated with the kernel, whose elements are real-valued functions on X. For example, in numerical analysis, it is often possible to reason that *f* possesses a certain number of derivatives, from which inclusion in certain Sobolev RKHSs can be deduced. The approach that we take is to identify the covariance function *k* with the kernel of an RKHS, denoted $H_{k}(X)$, in which *f* is contained. In particular, for any $k_{0}^{2}\in (0,\infty )$ the space reproduced by the kernel *k* in (3) consists of functions g:X→R of the form g(x)=μ+b(x)e(x) where μ∈R and $e\in H_{k_{e}}(X)$, and in the $k_{0}^{2}\to \infty$ limit the norm structure of $H_{k}(X)$ reduces to a semi-norm $|g{|}_{H_{k}(X)}\;:\;=\|x\mapsto (g(x)-g(0))/b(0){\|}_{H_{k_{e}}(X)}$ induced by the norm structure of $H_{k_{e}}(X)$; further background on RKHS can be found in Berlinet and Thomas-Agnan (2011). This construction results in a *conservative* prior GP, since with probability, one sample paths will be less regular than *f* when the RKHS is infinite-dimensional. However, there are several senses in which this approach to prior elicitation can be justified. First, it can be viewed as a form of ‘objective’ prior for GPs, in the sense that it is not intended to reflect prior belief but is rather intended to induce desirable behaviour in the posterior GP. Second, the choices that we make here will be justified through theoretical guarantees on both point estimation error (Section 2.4) and coverage of credible sets (Section 2.6). Third, the introduction of an additional scale estimator $\sigma_{n}^{2}[f]$ in (5) provides an opportunity to counteract the conservatism of the choice of *k* through the data-driven estimation of an appropriate scale for the credible sets in (7).

### 2.4 Higher-order convergence guarantees

The main technical contribution of this paper is to establish sufficient conditions under which the GRE point estimate $m_{n}[f](0)$ in (6) provides a more accurate approximation to the continuum limit f(0) compared to the highest fidelity approximation $f(x_{n})$ on which it is based. The analysis we present is based on local polynomial reproduction, similar to that described in Wendland (2004). However, our results differ from existing work in that they are adapted to the nonstationary kernel (3) and quantify the space-filling properties of a design $X_{n}$ using boxes, rather than balls or cones, since boxes are more natural for the domain $X\subseteq [0,\infty {)}^{d}$ and enable sharper control over the constants involved.

To state our results, we define the *box fill distance*  $\rho_{X_{n},X}$ as the supremum value of *ν* such that there is a box of the form [x,x+ν1] contained in X for which $X_{n}\cap [x,x+\nu 1]=\emptyset$. Define the constants $\gamma_{d}$ using the induction $\gamma_{d}\;:\;=2d(1+\gamma_{d-1})$ with base case $\gamma_{1}\;:\;=2$. Our first main result, whose proof is contained in Appendix B.4, concerns the finite-smoothness case, where polynomial-order acceleration can be achieved:

Theorem 2 **Higher-order convergence; finite smoothness**

Let $X=[0,1]\subset R^{d}$ and $X_{n}\subset X$. Let $X_{h}=[0,h1]$ and $X_{n}^{h}=\{hx\;:\;x\in X_{n}\}$ where h∈(0,1]. Assume that $f\in H_{k}(X)$, $b\in \pi_{r}(X)$ and $k_{e}\in C^{2s}(X\times X)$. Let $m_{n}^{h}[f](0)$ denote the point estimate (6) based on data $f(X_{n}^{h})$. Then there is an explicit *n*- and *h*-independent constant $C_{r,s}$, defined in the proof, such that

$$\underset{\text{extrapolation error}}{\underbrace{\left|f(0)-m_{n}^{h}[f](0)\right|}}\leq C_{r,s}\rho_{X_{n},X}^{s}|f{|}_{H_{k}(X)}\underset{\text{acceleration}}{\underbrace{h^{s}}}\;\underset{\text{original bound }}{\underbrace{\|b{\|}_{L^{\infty }(X_{h})}}}$$

whenever the box fill distance satisfies $\rho_{X_{n},X}\leq 1/(\gamma_{d}(r+2s))$.

To interpret the conclusion of Theorem 2, fix *n* to be large enough that the constraint on the box fill distance is satisfied and examine the convergence of $m_{n}^{h}[f](0)$ to f(0) as *h* is decreased. If the problem possesses no additional smoothness to exploit (i.e. s=0) then convergence is gated at the rate $\|b{\|}_{L^{\infty }(X_{h})}$ of the original numerical method, irrespective of the number *n* of data that are used to train the GP. On the other hand, if *f* is regular enough that the normalized error functional x↦(f(x)−f(0))/b(x) is an element of the RKHS $H_{k_{e}}(X)$ of an *s*-smooth kernel (implied by $|f{|}_{H_{k}(X)}<\infty$), then the $h^{s}$ factor provides acceleration of polynomial order *s* over the convergence rate of the original numerical method. To the best of our knowledge, these theoretical results are the first of their kind for convergence acceleration using GPs. Examples 5 and 8 illustrate cases in which our regularity assumptions are satisfied. For the reader’s convenience, we recall some standard examples of kernels and their associated smoothness properties in Appendix A.

Remark 3 **Sample efficiency compared to Richardson**

A notable feature of Richardson extrapolation is that, under appropriate regularity assumptions, acceleration of order *s* can be achieved using a dataset of size n=s+1 in dimension d=1. For example, if *f* is first-order accurate with $f(h)=f(0)+c_{1}h+O(h^{2})$, then the line that passes through data (h,f(h)) and (2h,f(2h)) has intercept 2f(h)−f(2h), which is equal to $2[f(0)+c_{1}h+O(h^{2})]-[f(0)+2c_{1}h+O(h^{2})]=$$f(0)+O(h^{2})$; an additional order of accuracy is gained. Our result is less sample-efficient, in the sense that n≥2r+4s data are in principle required, due to the constraint on the box fill distance in Theorem 2. However, we speculate that this lower bound on *n* is not tight, and we empirically confirm that order-*s* acceleration is observed at smaller sample sizes *n* in Examples 5 and 8.

On the other hand, if there is infinite smoothness to exploit, then we may consider increasing the value of *s* in Theorem 2 to obtain an arbitrarily fast convergence rate as h→0, albeit with an increasing number *n* of training points required for the bound to hold. This result goes beyond classical Richardson extrapolation, but is natural within the GP framework. Theorem 4, whose proof is contained in Appendix B.5, is obtained by carefully tracking the *s*-dependent constants appearing in Theorem 2:

Theorem 4 **Higher-order convergence; infinite smoothness**

In the setting of Theorem 2, assume further that $k_{e}\in C^{\infty }(X\times X)$ and that ${\text{sup}}_{x,y\in X}\sum_{|\beta |=2s}|\partial_{y}^{\beta }k_{e}(x,y)|\leq C_{k}^{2s}(2s)!$ for some constant $C_{k}$. Then, there exists an explicit *h*-independent constant $C_{n,r,s}$, defined in the proof, such that

$$\underset{\text{extrapolation error}}{\underbrace{\left|f(0)-m_{n}^{h}[f](0)\right|}}\leq C_{n,r,s}|f{|}_{H_{k}(X)}\underset{\text{acceleration}}{\underbrace{h^{\frac{1}{4\gamma_{d}\rho_{X_{n},X}}}}}\;\underset{\text{original bound}}{\underbrace{\|b{\|}_{L^{\infty }(X_{h})}}}$$

whenever the box fill distance satisfies $\rho_{X_{n},X}\leq \min\{1/(2\gamma_{d}(r+1)),$  $1/(2d^{1/2}\gamma_{d}e^{4d\gamma_{d}+1})\}$.

The derivative growth condition in the statement of Theorem 4 holds for most popular smooth kernels $k_{e}$, including the Gaussian kernel. The order of acceleration is now determined by the box fill distance, which reflects the general phenomenon that ‘more samples are required to exploit smoothness’ (Cabannes & Vigogna, 2024).

To assess the sharpness of our results, we first consider the problem of approximating derivatives using finite differences; a setting where extrapolation methods are routinely used (see Section 6.7 of Brezinski & Zaglia, 2013):

Example 5 **Higher-order convergence for finite difference approximation**

Consider numerical differentiation of a suitably regular function ψ:R→R. The *central difference method*

$$f(x)\;:\;=\frac{\psi (t+x)-\psi (t-x)}{2x},\;x>0,$$

is a second-order approximation to $\psi^{\prime }(t)$ for a given t∈R. To make use of our results, we set $b(x)=x^{2}$, from (3), and suppose that $\psi (t+x)=c_{0}+c_{1}x+c_{2}x^{2}+c_{3}(x)x^{3}$ for some $c_{0},c_{1},c_{2}\in R$ and some *x*-dependent coefficient $c_{3}(x)$. The normalized error is

$$e(x)=\frac{f(x)-f(0)}{b(x)}=\frac{\psi (t+x)-\psi (t-x)}{2x\cdot x^{2}}-\frac{\psi^{\prime }(t)}{x^{2}}=\frac{c_{3}(x)-c_{3}(-x)}{2},$$

so that the assumptions of Theorem 2 are satisfied when $x\mapsto c_{3}(x)$ and $x\mapsto c_{3}(-x)$ are elements of $H_{k_{e}}(X)$, and $k_{e}\in C^{2s}(X\times X)$ (the latter condition can be satisfied, for example, by taking $k_{e}$ to be either a Matérn kernel or a Wendland kernel with appropriate smoothness level; see Appendix A). As a test problem, consider $\psi (t)=\sin\;(10t)+1_{t>0}t^{s+4}$ with $\psi^{\prime }(0)=10$ the value to be estimated; this test problem is selected so that our assumptions hold precisely for an *s*-smooth kernel, as verified in Appendix B.6. The sample size n=5 was fixed and the initial design $X_{n}=\{0.2,0.4,0.6,0.8,1\}$ was scaled by a factor *h* to obtain a range of designs $X_{n}^{h}\subset (0,h]$. In these experiments, we work in 100 digits of numerical precision, so that rounding error can be neglected.

**Figure 2.**
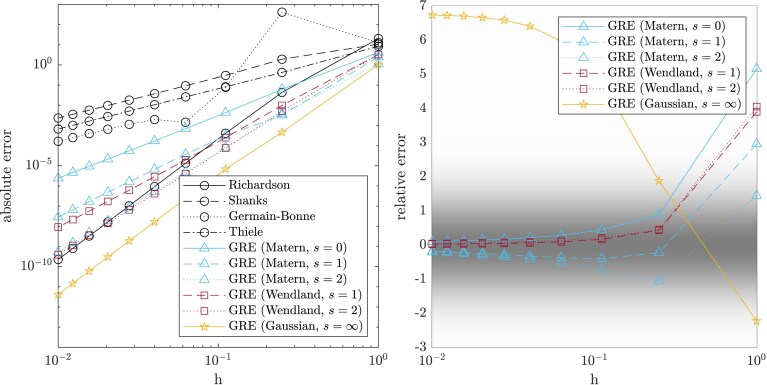
Accelerating the central difference method; Example 5. The left panel presents the absolute error $\left|f(0)-m_{n}^{h}[f](0)\right|$, while the right panel presents the relative error $(f(0)-m_{n}^{h}[f](0))/\sqrt{k_{n}[f](0,0)}$. Classical extrapolations methods (circles) were compared to our Gauss–Richardson Extrapolation (GRE) method, with either a Matérn (triangles), Wendland (squares), or Gaussian (stars) kernel. The true smoothness in this case is s=2, while the legend indicates the level of smoothness assumed by the kernel. Kernel length-scale parameters were set to ℓ=1 and the scale estimator $\sigma_{n}^{2}[f]$ proposed in Section 2.6 was used. Shaded regions in the right panel correspond to the density function of the standard normal.

Results for s=2 are reported in Figure 2, with the *absolute error*  $\left|f(0)-m_{n}^{h}[f](0)\right|$ plotted as a function of *h* in the left panel. These results reveal that the orders of acceleration predicted by our analysis are achieved, despite the sample size *n* being less than that required to fulfil the box fill distance requirement in Theorem 2. The GRE method demonstrated accuracy comparable to Richardson’s extrapolation method (and superior to other classical extrapolation methods) when the kernel was chosen to match the smoothness of the task at hand. Interestingly, the most accurate extrapolation was provided by GRE with the Gaussian kernel, despite this kernel being too smooth for the task at hand. The coverage of GRE credible intervals was also investigated, with the *relative error*  $(f(0)-m_{n}^{h}[f](0))/\sqrt{k_{n}[f](0,0)}$ plotted as a function of *h* in the right panel. It was found that credible intervals are asymptotically conservative in the case where a kernel with finite smoothness was used, in the sense that the relative error appeared to vanish in the h→0 limit. However, in the case of the Gaussian kernel, the credible intervals appeared to be asymptotically calibrated, in the sense that the relative error appeared to converge to a finite value (≈3) in the h→0 limit. Theoretical analysis of the GRE credible intervals is provided in Section 2.6.

Though they accurately describe the convergence acceleration provided by the GRE method, there are at least two apparent drawbacks with Theorems 2 and 4. The first is that these results require the error bound *b* to be a polynomial; this is an intrinsic part of our proof strategy, which is based on local polynomial reproduction, and cannot easily be relaxed. However, for applications in which a nonpolynomial error bound $\tilde{b}$ naturally arises, we may still be able to construct a polynomial error bound $b\in \pi_{r}(X)$ for some *r* that satisfies $\tilde{b}(x)\leq b(x)$ for all x∈X and enables the conclusion of Theorem 2 to be applied. The second limitation is that, for many iterative numerical methods that produce a convergent sequence of approximations to the continuum quantity f(0) of interest, there is not always the notion of a continuum of discretization parameters x that can be exploited in the GRE framework. This second issue can be elegantly addressed using the notion of an *s*-smooth extension, which we introduce next.

### 2.5 The generality of continua

Several numerical methods do not admit a continuum of discretization parameters x that can be exploited in the GRE method. For example, the conjugate gradient algorithm for approximating the solution to a linear system of equations produces a convergent sequence of approximations, but is in no sense continuously indexed. The aim of this section is to demonstrate that iterative methods, which produce a sequence of approximations converging to a limiting quantity of interest, do in fact fall within our framework. The idea, roughly speaking, is to construct a function *f* whose values $f(x_{n})$ on a convergent sequence, such as $x_{n}=1/n$, coincide with the approximation produced after *n* iterations of the numerical method. The challenge is to show that such a function *f* exists with sufficient regularity that the results of Section 2.4 can be applied. Our main tool is the idea of an *p*-smooth extension, which is the content of Proposition 6. Let $\min(z)\;:\;=\min\{z_{1},\ldots ,z_{d}\}$ for $z\in R^{d}$.

Proposition 6 ***p*-smooth extension**

Suppose that $C^{p}(X)\subset H_{k_{e}}(X)$ for some p∈N. Let $\left(x_{n}{)}_{n\in N}\subset X\setminus \{0\right\}$ be such that $x_{n+1}<x_{n}$ componentwise and $x_{n}\to 0$. Let $(y_{n}{)}_{n\in N}$ be a convergent sequence with limit $y_{\infty }$, such that the normalized errors $e_{n}\;:\;=(y_{n}-y_{\infty })/b(x_{n})$ satisfy $|e_{n}-e_{n+1}|/\min(x_{n}-x_{n+1}{)}^{p}\to 0$. Then there exists a function *f* such that $f(0)=y_{\infty }$, $f(x_{n})=y_{n}$ for each n∈N, and $|f{|}_{H_{k}(X)}<\infty$.

A polynomial expansion can be used to establish the preconditions of Proposition 6, as we illustrate in the following result:

Corollary 7 **Sufficient conditions for *p*-smooth extension in d=1**

Let $(x_{n},y_{n}{)}_{n\in N}\subset (0,\infty )\times R$ be such that $x_{n}$ converges monotonically to 0, with $(x_{n}^{\;p+1}-x_{n+1}^{\;p+1})(x_{n}-x_{n+1}{)}^{-p}\to 0$, $x_{n}^{\;p+2}(x_{n}-x_{n-1}{)}^{-p}\to 0$ and $y_{n}=y_{\infty }+C_{1}x_{n}^{r}+C_{2}x_{n}^{r+p+1}+O(x_{n}^{r+p+2})$ for some constants $y_{\infty },C_{1},C_{2}\in R$. Let $b(x)=x^{r}$. Then, the preconditions of Proposition 6 are satisfied.

The proof of both Proposition 6 and Corollary 7 can be found in Appendix B.7. The conditions on the sequence $(x_{n}{)}_{n\in N}$ in Corollary 7 are satisfied by, for example, sequences of the form $x_{n}=\frac{1}{n}$ and $x_{n}=\lambda^{-n}$ for any λ>1, which are the sort of expressions that routinely appear in error bounds. The overall approach is illustrated in Example 8, where a GP analogue of the classical Romberg method for numerical integration is derived.

Example 8 **GP Romberg methods**

**Figure 3.**
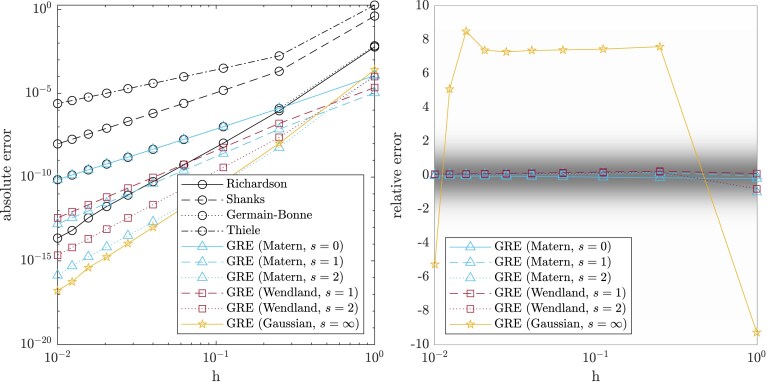
Accelerating the trapezoidal method to obtain a GP Romberg method; Example 8. The left panel presents the absolute error $\left|f(0)-m_{n}^{h}[f](0)\right|$, while the right panel presents the relative error $(f(0)-m_{n}^{h}[f](0))/\sqrt{k_{n}[f](0,0)}$. Classical extrapolations methods (circles) were compared to our GRE method, with either a Matérn (triangles), Wendland (squares), or Gaussian (stars) kernel. The true smoothness in this case is s=2, while the legend indicates the level of smoothness assumed by the kernel. Kernel length-scale parameters were set to ℓ=1. Shaded regions in the right panel correspond to the density function of the standard normal.

Romberg methods for numerical integration are classically obtained via Richardson extrapolation of the trapezoidal rule (Brezinski & Zaglia, 2013, Section 6.7); it is interesting to ask if a similar feat can be achieved with GRE. Let $\psi \in C^{2m+2}([0,1])$ and consider the trapezoidal rule $y_{n}\;:\;=\frac{1}{n}[\frac{\psi (0)}{2}+\psi (\frac{1}{n})+\ldots +\psi (\frac{n-1}{n})+\frac{\psi (1)}{2}]$. The Euler–Maclaurin summation formula implies that the error of the trapezoidal rule can be expressed as

$$y_{n}-\int_{0}^{1}\psi (t)\;dt=\sum_{i=1}^{m}\frac{B_{2i}}{(2i)!}x_{n}^{2i}\left(\psi^{\left(2i-1\right)}(1)-\psi^{\left(2i-1\right)}(0)\right)+\frac{B_{2m+2}}{(2m+2)!}x_{n}^{2m+2}\psi^{\left(2m+2\right)}(\beta_{n})$$

for some $\beta_{n}\in [0,1]$, where $x_{n}=\frac{1}{n}$ and $B_{k}$ are the Bernoulli numbers. As a test problem, consider $\psi (t)=\sin\;(10t)+t^{2}$, for which we can apply Corollary 7 with $b(x)=x^{2}$, r=2 and p=3. Thus there exists a function *f* that agrees with the trapezoidal rule on $(x_{n}{)}_{n\in N}$ and satisfies the preconditions of Theorem 2 for a kernel $k_{e}$ with smoothness up to s=2; see Appendix A. Empirical results in Figure 3 verify that we are indeed able to gain an additional s=2 convergence orders over the original trapezoidal rule, akin to Romberg integration, using our GRE method. Here, the sample size n=5 was fixed and the initial design $X_{n}=\{1,\frac{1}{2},\frac{1}{4},\frac{1}{8},\frac{1}{16}\}$ was scaled by a factor *h* to obtain a range of designs $X_{n}^{h}\subset (0,h]$. The accuracy of the GRE point estimator and the coverage of the GRE credible interval demonstrate similar behaviour to that observed in Example 5.

These results extend the applicability of GRE to settings where components of the discretization parameter vector x could take values in any infinite set. For example, in standard implementations of the finite-element method for numerically solving partial differential equations one has a continuous parameter, characterizing the width of a triangular mesh, a discrete parameter, characterizing the number of cubature nodes used to integrate against each element, and another discrete parameter, specifying the number of iterations of a conjugate gradient method to solve the resulting linear system. The resulting mixture of continuous and discrete discretization parameters x falls within the scope of our GRE method.

### 2.6 Uncertainty quantification

An encouraging observation from Examples 5 and 8 was that the GRE credible intervals were not asymptotically over-confident as h→0. The aim of this section is to explain how the scale parameter $\sigma^{2}$ in (3), which controls the size of credible intervals $C_{\alpha }[f]$ in (7), was actually estimated, and to rigorously prove that asymptotic over-confidence cannot occur when our proposed estimator $\sigma_{n}^{2}[f]$ is used.

The most standard approach to kernel parameter estimation is maximum (marginal) likelihood, but in GRE we do not have a valid likelihood due to taking the improper $k_{0}^{2}\to \infty$ limit. Instead, we motivate a particular estimator $\sigma_{n}^{2}[f]$ using asymptotic guarantees for the associated credible interval. Specifically, we advocate the estimator


(8) $$\sigma_{n}^{2}[f]\;:\;=\frac{|m_{n}[f]{|}_{H_{k}(X)}^{2}}{n}=\frac{1}{n}\left[\;f(X_{n}{)}^{⊤}K_{b}^{-1}f(X_{n})-\frac{(1^{⊤}K_{b}^{-1}f(X_{n}){)}^{2}}{1^{⊤}K_{b}^{-1}1}\right],$$

which takes the same form as the maximum-likelihood estimator that we would have obtained had we not taken the $k_{0}^{2}\to \infty$ limit, but with the semi-norm $|m_{n}[f]{|}_{H_{k}(X)}$ in place of the conventional norm on $H_{k}(X)$. This choice is supported by the following asymptotic result, whose proof is contained in Appendix B.8:

Proposition 9 **Asymptotic over-confidence is prevented**

In the setting of Theorem 2, suppose that s≥1 and that ${\text{lim}}_{x\to 0}b(x{)}^{-1}(f(x)-f(0))\neq 0$ (i.e. we have a sharp error bound). Let $m_{n}^{h}[f](0)$ and $k_{n}^{h}[f](0,0)$ denote the conditional mean and variance in (6), based on data $f(X_{n}^{h})$ and the estimator in (8). Then

$$\underset{h\to 0}{\lim\;\sup}\;\frac{\left|f(0)-m_{n}^{h}[f](0)\right|}{\sqrt{k_{n}^{h}[f](0,0)}}<\infty$$

whenever the box fill distance $\rho_{X_{n},X}$ is sufficiently small.

In other words, the width $\sqrt{k_{n}[f](0,0)}$ of the credible interval cannot vanish asymptotically faster than the actual absolute error $\left|f(0)-m_{n}[f](0)\right|$. Though this result does not guarantee that credible intervals are the ‘right size’ per se, there is no randomness in the data-generating process f(x) and thus standard statistical notions of coverage, or ‘right size’, cannot be directly applied (see Karvonen et al., 2020). In practice, we have already seen empirical evidence that the credible sets (7) are appropriately conservative; an arguably predictable consequence of the conservative GP prior discussed in Section 2.3. Note that the conclusion of Proposition 9 also holds when the stronger hypotheses of Theorem 4 are assumed. However, the result assumes that a kernel with appropriate smoothness is used; it does not explain the behaviour of GRE with the Gaussian kernel observed in Examples 5 and 8, since in that case the Gaussian kernel was formally misspecified.

Assured that our credible intervals are in a sense meaningfully related to the actual error, we can now proceed to exploit this measure of uncertainty for experimental design.

### 2.7 Optimal experimental design

One of the main engineering challenges associated with the simulation of continuum mathematical or physical phenomena is the numerical challenge of simultaneously controlling all sources of discretization error, to ensure the output f(x) remains close in some sense to f(0), the continuum quantity of interest. In practice, one might explore the sensitivity of the simulator output f(x) to small changes in each discretization parameter $x_{i}$ in turn, to heuristically identify a global setting $x_{\mathrm{hi}-\mathrm{fi}}$ which is then fixed for the lifetime in which the simulator is used. It seems remarkable that more principled methodology has not yet been developed, and we aim to fill this gap by formulating *optimal experimental design* within the GRE framework.

The accuracy of the point estimator (6) will depend crucially on the locations at which the GP has been trained. Section 2.6 established that the conditional variance is meaningfully related to estimation accuracy, with the advantage that it can be explicitly calculated. This motivates the following cost-constrained optimization problem


(9) $$\underset{X\subset D}{\text{arg max}}\;1^{⊤}K_{b}^{-1}1\;\text{s.t.}\;\;\sum_{x\in X}c(x)\leq C,$$

where D⊆X denotes the set of feasible simulations being considered, $K_{b}$ is the matrix with entries $k_{b}(x_{i},x_{j})$, $x_{i},x_{j}\in X$, the map c:D→R quantifies the cost associated with obtaining simulator output f(x), and *C* denotes the total computational budget. This numerical analysis-informed objective $1^{⊤}K_{b}^{-1}1$ is inversely proportional to the GRE posterior variance (6) when the scale parameter *σ* is fixed, rather than estimated (since a priori we do not suppose data have been obtained from which *σ* could be estimated). This optimization does not enforce a particular training sample size *n*, it just constrains the total computational cost. As such, (9) represents a challenging optimization problem, with both the number *n* of experiments in the optimal design, and the optimal experiments $X=\{x_{i}{\}}_{i=1}^{n}$ themselves, to be determined. To proceed, we consider a finite set D of candidate experiments and then use brute force to search for an optimal design restricted to this candidate set, but we note that better search strategies can surely be developed.

Example 10 **Optimal experimental design in d=1**

**Figure 4.**
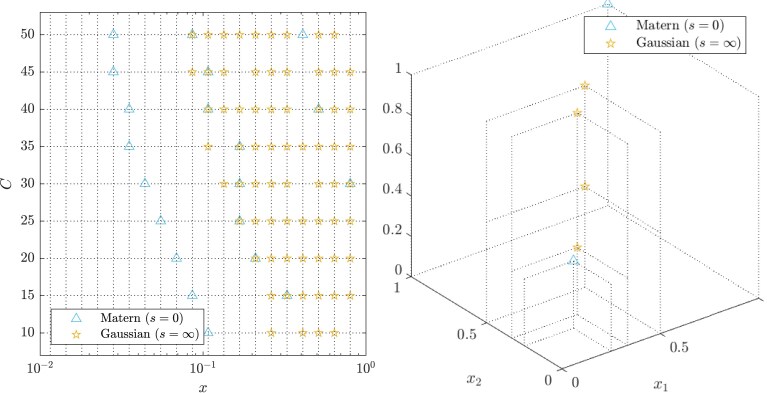
Optimal experimental designs were computed, for varying total computational budgets *C*, using either a Matérn (triangles; s=0) or Gaussian (stars; s=∞) kernel. Left: The setting of Example 10, with candidate states shown as vertical dotted lines on the plot. Right: An illustration of experimental design in dimension d=3, with dotted lines used to indicates the coordinates of the states that were selected.

Consider a first-order numerical method with linear cost, so that b(x)=x and $c(x)=x^{-1}$, an example of which would be the classical forward Euler method. For illustration, we take $k_{e}$ to be either a Matérn kernel (s=0) or the Gaussian kernel (s=∞), in each case with length-scale ℓ=1 fixed. The total computational budget *C* was varied and optimal designs *X* were computed with elements constrained to a size 20 grid D; results are shown in Figure 4. In the case of a rough kernel, like the Matérn kernel, a greedy/exploitative strategy of assigning all compute power to the highest resolution experiment seems optimal. Since we are working only with a discrete set of experiments, there is a small residual computational budget that is allocated to one or two further cheap experiments. For the Gaussian kernel, the optimal strategy is less greedy, with optimal designs involving more experiments, indicating that the greater smoothness is being leveraged to improve the accuracy of GRE.

In practice, a small number of preliminary simulations should be used to estimate appropriate length-scale parameters ℓ for the covariance kernel. Such parameter estimation becomes more critical in the multivariate setting, illustrated in the right panel of Figure 4, since the simulator output f(x) may be more or less sensitive to different components of x; in Section 3, a practical workflow is presented.

Remark 11 **Trivial solution for iterative methods**

In Section 2.5, we discussed the scenario where data are generated along a sequence $(x_{n}{)}_{n\in N}$ by an iterative method, which first produces $f(x_{1}),\ldots ,f(x_{n-1})$ en route to producing the final output $f(x_{n})$. In this scenario, the cost of computing $f(x_{1}),\ldots ,f(x_{n})$ is simply $c(x_{n})$, in which case computing as many iterations as possible is optimal in the sense of (9).

The methodology just presented systematizes the often *ad-hoc* process of selecting appropriate fidelities on which simulator output is computed, in a manner that is specifically tailored to improving the accuracy of our GRE method. Sequential experimental design strategies can also be developed, but were not pursued. The remainder of this section deals with three important generalizations of the GRE method; the case where convergence orders are unknown and must be estimated (Section 2.8), the case of multivariate simulator output (Section 2.9), and the case where the simulator contains additional degrees of freedom (Section 2.10).

### 2.8 Extension to unknown convergence order

The practical application of extrapolation methods does not necessarily require access to an explicit error bound, as several procedures have been developed to automatically identify a suitable method from a collection of extrapolation methods (which could correspond to different assumed convergence orders, or different classes of extrapolation method). A representative approach, called *automatic selection* (Delahaye, 1981), is based on the idea that small changes $S_{n+1}-S_{n}$ between consecutive iterates is a useful proxy for the convergence rate of an extrapolation method $(S_{n}{)}_{n\in N}$. Another approach is to linearly combine estimates produced by a collection of extrapolation methods, called a *composite sequence approach* (Brezinski, 1985). From our statistical standpoint, these methods bear a respective semblance to model selection and model averaging. Pursuing a statistical perspective on extrapolation, here we consider maximum (marginal) likelihood as a default for selecting an appropriate GP prior model for GRE. The $k_{0}^{2}\to \infty$ limit taken in Section 2.2 means that we do not have a proper likelihood, so instead, we identify and maximize an appropriate *quasi*-likelihood. Our justification is twofold, namely (1) our quasi-likelihood is directly analogous to the standard GP likelihood, and (2) we provide analysis below that demonstrates the consistency of maximum quasi-likelihood for estimation of convergence order in the GRE framework.

To formulate the main result of this section, we suppose we have a vector $r\in R^{p}$ that parametrizes the error bound $b_{r}\;:\;X\to [0,\infty )$, with the interpretation that increasing the value of any of the components of r corresponds to faster convergence of the error bound $b_{r}(x)$ to 0 as x→0. Specifically, we call a class of error bounds *monotonically parametrized* if, for all $r_{1}<r_{2}$, we have


$$\underset{r\leq r_{1}}{\inf}\underset{x\to 0}{\text{lim}}\;\frac{b_{r_{2}}(x)}{b_{r}(x)}=0.$$

This is not a restriction per se, as we are free to choose how $b_{r}$ is parametrized, but an assumption of this kind is required to enable the following result to be rigorously stated. Examples of monotonically parametrized error bounds include $b_{r}(x)=x_{1}^{r_{1}}+\ldots +x_{d}^{r_{d}}$ and $b_{r}(x)=x_{1}^{r_{1}}\cdots x_{d}^{r_{d}}$, which are the sort of expressions that routinely appear in error bounds. The proof of the following result can be found in Appendix B.9:

Proposition 12 **Estimation using maximum quasi-likelihood**

Let $X_{n}^{h}=\{hx\;:\;x\in X_{n}\}$. Suppose that $f\in H_{k}(X)$ holds when *k* in (3) is based on the monotonically parametrized bound $b_{r_{0}}(x)$ for some $r_{0}\geq 0$. Let $K_{b_{r},h}$ denote the matrix with entries $k_{b_{r}}(hx_{i},hx_{j})$, where the dependence of this matrix on both *h* and r has now been emphasized, relative to the notation $K_{b}$ introduced in Section 2.2. Then any maximizer $r_{n}^{h}[f]\in {\text{arg max}}_{r\geq 0}L_{n}^{h}(r)$ of the log-quasi (marginal) likelihood

(10) $$L_{n}^{h}(r)\;:\;=-f(X_{n}^{h}{)}^{⊤}K_{b_{r},h}^{-1}f(X_{n}^{h})+\frac{(1^{⊤}K_{b_{r},h}^{-1}f(X_{n}^{h}){)}^{2}}{1^{⊤}K_{b_{r},h}^{-1}1}-\log\;\text{det}K_{b_{r},h}$$

satisfies ${\text{lim inf}}_{h\to 0}r_{n}^{h}[f]\geq r_{0}$.

The first two terms in (10) correspond to the (square of the) semi-norm $|m_{n}^{h}[f]{|}_{H_{k}(X)}$, which is the analogue of the usual $\|m_{n}^{h}[h]{\|}_{H_{k}(X)}$ term that would appear in the likelihood had we not taken the $k_{0}^{2}\to \infty$ limit; this justifies the interpretation of (10), up to constants, as a quasi-likelihood. The one-sided conclusion of Proposition 12 may be surprising at first, but this is in fact the strongest result that can be expected. Indeed, the statement that $f(x)-f(0)=O(b_{r_{0}}(x))$ does not rule out the possibility that the error f(x)−f(0) decays *faster* than $b_{r_{0}}(x)$, and in this case we would expect the estimator $r_{n}^{h}[f]$ to adapt to the actual convergence order. The experiments that we report in Section 3 used maximum quasi (marginal) likelihood whenever convergence orders and/or kernel length-scale parameters were estimated.

Remark 13 **When to extrapolate?**

The error bounds $b_{r}(x)$ describe asymptotic behaviour as x→0 only, and it is reasonable to ask whether given data $\{f(x_{i}){\}}_{i=1}^{n}$ are collected from a regime where such asymptotics are actually observed. Though we do not develop it further in this work, our statistical perspective enables goodness-of-fit testing and related techniques to assess the suitability of given data for being extrapolated.

### 2.9 Generalization to multidimensional output

Until this point, we have considered the continuum quantity of interest f(0) to be scalar-valued. Oftentimes, however, we are interested in quantities $\{f(0,t){\}}_{t\in T}$ that are vector- or function-valued depending on the nature of the index set T. The E-algorithm that we described in Section 1 has been extended to finite-dimensional vector-valued output; see Chapter 4 of Brezinski and Zaglia (2013) for detail. A possible advantage of the GP-based approach taken in GRE is that it does not impose any mathematical structure on T beyond this being a set, making extension of the methodology to function-valued output straight-forward.

To extend our methodology to multivariate output, let f:X×T→R be such that $\{f(0,t){\}}_{t\in T}$ is the continuum quantity of interest and f(x,t) is a numerical approximation to f(0,t). For example, f(0,t) may represent the solution to an ordinary differential equation at time *t*, while f(x,t) may represent an approximation to this solution obtained using a Runge–Kutta method, with *x* being the error tolerance of the Runge–Kutta method. To improve presentation, we will assume that f(x,t)−f(0,t)=O(b(x))  *uniformly* over t∈T, but t-dependent error bounds could also be considered with additional notational overhead. Our original covariance function (3) can be generalized to


(11) $$k((x,t),(x^{\prime },t^{\prime }))=\sigma^{2}\left[k_{0}^{2}+b(x)b(x^{\prime })k_{e}(x,x^{\prime })\right]k_{T}(t,t^{\prime }),\;x,x^{\prime }\in X,t,t^{\prime }\in T,$$

where to exploit tractable computation that results from this tensor product kernel, we have assumed a tensor product kernel and will assume that data $X_{n}=\{(x_{i},t_{j}){\}}_{i=1\;j=1}^{n_{1}\;n_{2}}$ are obtained on a Cartesian grid ($n=n_{1}n_{2}$). That is, with the data appropriately ordered, we have the Kronecker decomposition $K=K_{X}⊗K_{T}$, where $K_{X}$ is the matrix with entries $k_{0}^{2}+b(x_{i})b(x_{j})k_{e}(x_{i},x_{j})$, and $K_{T}$ is the matrix with entries $k_{T}(t_{i},t_{j})$. Then, analogous calculations to those detailed in Appendix B.2, which we present in Appendix B.10, show that for values of $t,t^{\prime }$ contained in the dataset, the conditional mean and covariance function in the $k_{0}\to \infty$ limit are


mn[f](x,t)={kb(x)⊤Kb−1+[1−kb(x)⊤Kb−11]1⊤Kb−11⊤Kb−11}⊗[kT(t)KT−1]f(Xn)kn[f]((x,t),(x′,t′))=σn2[f]{kb(x,x′)kT(t,t′)−[kT(t)⊤KT−1kT(t′)](1−μτnτn+1)×[kb(x)⊤Kb−1kb(x′)−[kb(x)⊤Kb−11−1][kb(x′)⊤Kb−11−1]⊤1⊤Kb−11]},

where $k_{T}(t)$ is the vector with entries $k_{T}(t_{i},t)$. For values of $t,t^{\prime }$ not contained in the training dataset, the conditional covariance does not have a finite limit; a proper prior should be used if off-grid prediction in the t-domain is required. Further details on the multivariate setting are deferred to Section 3, where the approach is explored in the context of predicting temporal output from a cardiac model.

### 2.10 Incorporating additional degrees of freedom

The final methodological extension that we consider is the case where $f_{\theta }(x)$ additionally depends on one or more degrees of freedom θ∈Θ; a setting where emulation or MFM methods are routinely used (cf. Section 1). The proposed GRE method can be applied in this context by viewing $f_{\theta }(0)$ as a simulator with multidimensional output $\{f(0,\theta ){\}}_{\theta \in \Theta }$ and then applying the methodology described in Section 2.9 with θ, rather than t, indexing the output of this extended model. Since the required calculations are identical, we do not dwell any further on this point.

This completes our exposition of the GRE method. Next, we next turn to a cardiac modelling case study, where the usefulness of the methodology is evaluated.

## 3 Case study: cardiac modelling

**Figure 5.**
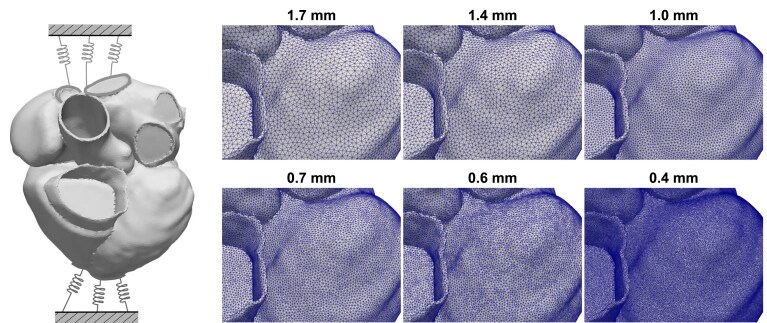
Cardiac model: Left: Schematic indicating the veins and the apical region where spring boundary conditions were applied. Right: A subset of the mesh resolutions used in this case study. The finest resolution required $3\times 10^{7}$ finite elements to be used.

The cardiac model $f_{\theta }(x)$ that we consider in this section is a detailed numerical simulation of a single heart beat^1^ (Strocchi et al., 2023). The simulation is rooted in finite element methods that require both a spatial ($x_{1}$) and a temporal ($x_{2}$) discretization level to be specified; of these, the spatial discretization is the most critical, due to the $O(x_{1}^{-3})$ cost associated with the construction of a suitable triangulation of the time-varying 3-dimensional volume of the heart; see Figure 5. The computational cost c(x) is measured in real computational time (seconds) and comprises the *setup time*, *assembly time* (the time taken to assemble linear systems of equations), and the *solver time* (the time taken to solve linear systems of equations), with assembly time the main contributor to total computational cost. To achieve a clinically acceptable level of accuracy, it is typical for a simulation $f_{\theta }(x_{\mathrm{default}})$ to be performed with $x_{\mathrm{default}}\approx (0.4\;\text{mm},2\;\text{ms})$, at a cost $c(x_{\mathrm{default}})\approx 1.5\times 10^{4}$ s (around ≈4 hr) for a single heart beat.^2^ This poses severe challenges to the scientific use of such models, with super-computing resources required to ascertain whether there are values of scientific parameters θ for which observed data are consistent with model output (Strocchi et al., 2023). These challenges directly motivated the development of GRE, and the remainder of this paper is dedicated to exploring the value of extrapolation methods in this context. Extrapolation of the cardiac model output represents a much greater challenge compared to extrapolation for the examples considered in Section 2, due to the nonlinear physics being simulated. Since our focus in this paper is not on inference for θ, these degrees of freedom were fixed to physically realistic values based on previous analyses (Strocchi et al., 2020, 2023), with all further details on the construction of the cardiac model reserved for Appendix C.

Section 3.1 sets out a practical workflow for using the GRE method, that focuses on the multidimensional setting where both convergence orders and kernel length-scale parameters are to be estimated. The performance of GRE is then investigated for both scalar-valued (Section 3.2) and multivariate (Section 3.3) continuum quantities of interest.

### 3.1 A proposed general workflow

The sophistication of the cardiac model renders analytical derivation of convergence orders essentially impossible, so to proceed these orders must be estimated. However, the computational cost of simulating from the model means that data from which convergence orders can be estimated are necessarily limited. This motivates us to propose the following pragmatic workflow, which we present for a general model f(x) and which scales in a reasonable way with the number *d* of components of x that can be varied. This workflow requires the user to specify a lo-fi setting $x_{\mathrm{lo}-\mathrm{fi}}$ as a starting point, together with a means to predict the computational cost c(x) of simulating f(x), and a total computational budget *C*:

1. For each fidelity parameter $x_{i}$, i=1,…,d:
  - Simulate f(x) for a range of values of $x_{i}$, with all of the other components x held fixed to their values in $x_{\mathrm{lo}-\mathrm{fi}}$.
  - Fit a univariate numerical analysis-informed GP model (4), (5) to these data, assuming an error bound of the form $b(x_{i})=x_{i}^{r_{i}}$, where the scale estimate ${\hat{\sigma }}_{i}$ from Section 2.6 is used, and where the convergence order $r_{i}$, the kernel smoothness $s_{i}$, and the kernel length-scale parameter $\ell_{i}$ are simultaneously estimated using quasi maximum likelihood, as explained in Section 2.8.
2. Construct a tensor product covariance model $k_{e}(x,x^{\prime })=k_{e}(x_{1},x_{1}^{\prime };\ell_{1})\ldots k_{e}(x_{d},x_{d}^{\prime };\ell_{d})$ and posit the overall error bound $b(x)={\hat{\sigma }}_{1}x_{1}^{r_{1}}+\ldots +{\hat{\sigma }}_{d}x_{d}^{r_{d}}$. Then, perform experimental design as described in Section 2.7, with computational budget *C*. Denote the optimal design $X_{n}$.
3. Simulate f(x) for each $x\in X_{n}$ and return the GRE conditional mean (6) as the final approximation to f(0).

Several remarks are in order: First, it is assumed that the Step 1 incurs negligible cost relative to the total computational budget; the precise interpretation of this assumption will necessarily be context-dependent. Second, the additive form for b(x) is appropriately conservative, in the sense that *all* components of x must be small to control this bound. One could go further and compare the performance of GPs based on alternative form of b(x), for example, with interaction terms included, selecting among such models using maximum quasi-likelihood, but for the present purposes the additive form of b(x) is preferred since it is compatible with the independent estimation of convergence orders $r_{i}$ in Step 1. Third, the independent estimation of $\left(r_{i},s_{i},\ell_{i}\right)$ for each i=1,…,d can be performed using brute-force search over a 3-dimensional grid to maximize the quasi-likelihood, whereas simultaneous estimation of all kernel parameters would be both statistically and computationally difficult. The full workflow is demonstrated on our cardiac case study, next.

### 3.2 Approximation of scalar quantities of interest

The first part of our case study concerned the approximation of physiologically interpretable scalar-valued quantities of interest. These were the minimum volume of the left and right ventricles and atria, the maximum volume during ventricular contraction for the left and right atria, and the time taken for the ventricles to contract in total capacity by one-half; a total of seven test problems for GRE.

Though the computational time $c(x_{\mathrm{default}})$ is substantial, in this case, study parallel computation resources can be exploited. The main computational constraint that we work under here is that we will only run experiments for which $c(x)\leq c(x_{\mathrm{default}})$ within our GRE method. To circumvent the complication of predicting computational times before experiments are performed, for this case study, a discrete set of experiments were performed at the outset and their times recorded. Since the continuum limit f(0) is intractable, we additionally computed a reference solution $f(x_{\mathrm{hi}-\mathrm{fi}})$ with $x_{\mathrm{hi}-\mathrm{fi}}=(0.4\;\text{mm},1\;\text{ms})$ and in what follows we assess how well the GRE point estimate $m_{n}[f](x_{\mathrm{hi}-\mathrm{fi}})$ approximates $f(x_{\mathrm{hi}-\mathrm{fi}})$. The central question here is whether the workflow proposed in Section 3.1 can provide more accurate approximation of $f(x_{\mathrm{hi}-\mathrm{fi}})$ compared to $f(x_{\mathrm{default}})$, and if so what computational budget is required. To the best of our knowledge, there do not exist comparable methodologies for this task; methods such as emulation and MFM are not applicable when θ is fixed, and classical extrapolation methods were not developed with multivariate x in mind.

**Figure 6.**
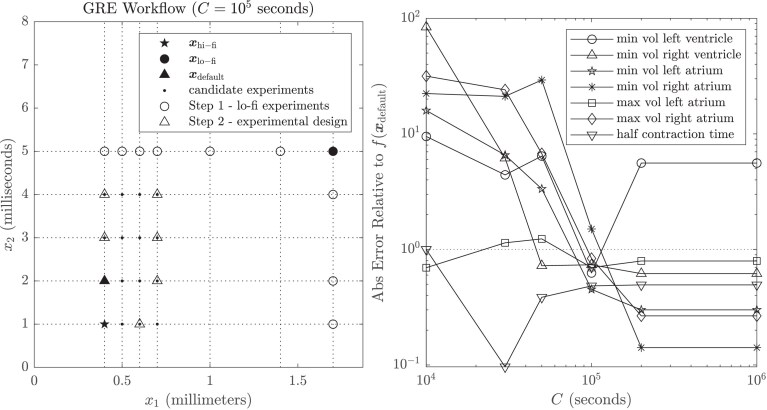
Scalar quantities of interest from the cardiac model. Left: The workflow, illustrated. In Step 1, the effect of varying each component of x in turn is explored, with all other components fixed equal to their value in $x_{\mathrm{lo}-\mathrm{fi}}$. This facilitates the construction of a multivariate Gaussian process model for use in Step 2, where experimental design is performed (here shown for a computational budget of $C=10^{5}$ s). For assessment purposes we aim to predict $f(x_{\mathrm{hi}-\mathrm{fi}})$ as a ground truth, but in practice the goal is to predict f(0). Right: For each of the seven scalar quantities of interest associated with the cardiac model we display the ratio of the absolute error $\left|f(x_{\mathrm{hi}-\mathrm{fi}})-m_{n}[f](x_{\mathrm{hi}-\mathrm{fi}})\right|$ of the GRE method and the absolute error $\left|f(x_{\mathrm{hi}-\mathrm{fi}})-f(x_{\mathrm{default}})\right|$ of the default approximation, as a function of the total computational budget *C*.

The workflow is illustrated in the left panel of Figure 6. The lo-fi setting was $x_{\mathrm{lo}-\mathrm{fi}}=$  (1.7mm,5ms). The convergence orders $r_{1}$, $r_{2}$ were selected from {0.5,1,2}, the smoothness parameters $s_{1}$, $s_{2}$ were selected from {0,1,2}, and the length-scales $\ell_{1}$, $\ell_{2}$ were selected using grid search, all estimated simultaneously using maximum quasi-likelihood. Experimental designs were computed based on a candidate set of experiments, each of which incurs a cost no greater than $c(x_{\mathrm{default}})$, indicated by dots in the left panel of Figure 6. Results for the seven test problems are shown in the right panel of Figure 6, where it is observed that the GRE point estimator provides a generally better approximation to $f(x_{\mathrm{hi}-\mathrm{fi}})$ compared to $f(x_{\mathrm{default}})$ when the computational budget *C* reaches or exceeds $10^{5}$. The optimal design for approximating the minimum volume of the left ventricle is depicted in the left-hand panel of Figure 6 for a computational budget $C=10^{5}$; the design supplements $x_{\mathrm{default}}$ with 6 additional simulations of lower cost, analogous to a classical extrapolation method but here generalized to the multivariate context. Note that for *C* exceeding $2\times 10^{5}$ the optimal design becomes saturated, containing all experiments in the candidate set. That GRE should perform worse than $f(x_{\mathrm{defult}})$ at small computational budgets is not surprising given that all convergence orders $r_{i}$, smoothnesses $s_{i}$, and length-scales $\ell_{i}$ are estimated from the small lo-fi training dataset, and these values largely determine the output of GRE in the absence of a sufficient number of experiments in the training dataset $X_{n}$. However, for a sufficiently large computational budget, it is encouraging to see that information from the experiments in $X_{n}$, each of which cost no greater than $c(x_{\mathrm{default}})$, is exploited in GRE to achieve more accurate estimation for 6 of the 7 scalar quantities of interest.

### 3.3 Approximation of temporal model output

**Figure 7.**
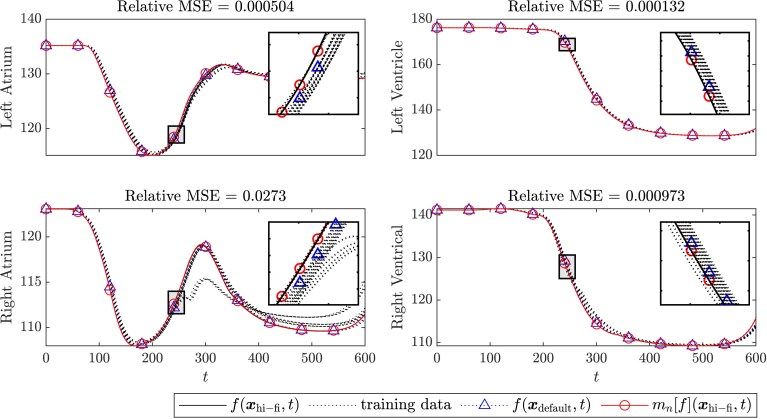
Temporal quantities of interest from the cardiac model. For each of the four temporal quantities of interest associated with the cardiac model we display the approximations produced at the different spatial and temporal resolutions in the training dataset $X_{n}$, together with the ground truth $f(x_{\mathrm{hi}-\mathrm{fi}})$ (solid), the default approximation $f(x_{\mathrm{default}},t)$ (triangles), and the approximation $m_{n}[f](x_{\mathrm{hi}-\mathrm{fi}},t)$ from Gauss–Richardson extrapolation (circles). The ratio of the mean square error $\int [f(x_{\mathrm{hi}-\mathrm{fi}},t)-m_{n}[f](x_{\mathrm{hi}-\mathrm{fi}},t){]}^{2}\;dt$ of the GRE method and the mean square error $\int [f(x_{\mathrm{hi}-\mathrm{fi}},t)-f(x_{\mathrm{default}},t){]}^{2}\;dt$ of the default method is also reported.

The scalar quantities of interest considered in Section 3.2 are summary statistics obtained from 4-dimensional temporal model output of the form f(x,t), where here *t* is a time index ranging from 0 to 600 ms and the components of f refer to the volumes of the atria and ventricles. It is, therefore, interesting to investigate whether these temporal outputs can be directly approximated, providing four test problems for the methodology described in Section 2.9. Here, for simplicity, we fixed the discrete values $s_{1}$, $s_{2}$, $r_{1}$, and $r_{2}$, the median of the values estimated in Section 3.2, and we fixed the continuous values ${\hat{\sigma }}_{1}$, ${\hat{\sigma }}_{2}$, $\ell_{1}$, and $\ell_{2}$ to the mean of the values estimated in Section 3.2. The length-scale for the kernel $k_{T}$ was set equal to the length of the time series itself. The computational budget was fixed to $C=2\times 10^{5}$, so that our experimental design is saturated, but recall that no individual experiment in this design had cost exceeding $c(x_{\mathrm{default}})$. Full results are displayed in Figure 7. In each case, the approximation produced by GRE achieves lower mean square error relative to $f(x_{\mathrm{default}},t)$. Taken together with the results in Section 3.2, these results are an encouraging and pave the way for subsequent investigations and applications of GRE.

## 4 Discussion

This paper introduced a probabilistic perspective on extrapolation, presenting a framework in which classical extrapolation methods from numerical analysis and modern MFM are unified. One approach was developed in detail, which we termed GRE. The GRE method facilitates simultaneous convergence acceleration and uncertainty quantification, and unlocks experimental design functionality for optimization over the set of fidelities at which simulation is performed. The end result is a methodology that allows a practitioner to arrive, in a principled manner, at fidelities $\{x_{i}{\}}_{i=1}^{n}$ such that the associated simulator outputs $\{f(x_{i}){\}}_{i=1}^{n}$ can be combined to produce an approximation to the continuum quantity f(0) that is typically more accurate than a single hi-fi simulation run at a comparable computational cost. A cardiac modelling case study provided an initial positive proof-of-concept, but further case studies—involving different types of computer model—will be required to comprehensively assess GRE; we aim to undertake domain-specific investigations in future work.

Several methodological extensions to this work can be envisaged, such as considering alternative regression models to GPs, developing theory and methodology for the more challenging cases where the regression model is misspecified and computational costs needs to be predicted, and extending the experimental design methodology to include additional degrees of freedom θ, which are often present in a mathematical model. In addition, and more speculatively, it would be interesting to explore modern computational tasks, such as the super-resolution task in deep learning, for which extrapolation methods have yet to be exploited.

## Supplementary Material

qkae098_Supplementary_Data

## Data Availability

The data that support the findings of this study are available on GitHub.

## Footnotes

The simulation is usually run until a steady state is reached before reading off quantities of interest, at a substantial increase to the overall computational cost. For the present purpose, we removed components from the model that required multiple heart beats to reach a steady state, and simulated only a single heart beat.

Simulations for this case study were performed on ARCHER2, a UK national super computing service (https://www.archer2.ac.uk/). Each simulation involved 512 CPUs operating in parallel, so that simulation of one heart beat using setting $x_{\mathrm{default}}$ required ≈4×512 CPU hours in total.
