# Supplementary material for: Probabilistic Richardson extrapolation
Source: J R Stat Soc Series B Stat Methodol. 2024 Dec 26;87(2):457–79. doi: 10.1093/jrsssb/qkae098 (PMC11985099; doi:10.1093/jrsssb/qkae098)
Supplement: qkae098_Supplementary_Data [file qkae098_supplementary_data.zip › supplement.pdf]

Appendices

A Kernels and Smoothness Spaces **1**

B Proofs of Results in the Main Text **2**

B.1 Sample Path Properties of Numerical Analysis-Informed GPs 2

B.2 Derivation of the 'Objective' Prior Limit 4

B.3 Technical Results on Polynomial Reproduction 5

B.4 Proof of Theorem 2 7

B.5 Proof of Theorem 4 9

B.6 Verifying the Assumptions for Example 5 10

B.7 Proof of Proposition 6 and Corollary 7 11

B.8 Proof of Proposition 9 11

B.9 Proof of Proposition 12 13

B.10 Calculations for Multidimensional Output 14

C Details for the Cardiac Model **15**

A Kernels and Smoothness Spaces

This appendix contains definitions for the kernels $k_e : \mathcal{X} \times \mathcal{X} \to \mathbb{R}$, on a bounded set $\mathcal{X} \subset \mathbb{R}^d$, referred to in the main text, together with details about the smoothness of elements in the Hilbert spaces $\mathcal{H}_{k_e}(\mathcal{X})$ that are reproduced. All kernels k_e that we discussed take the radial form

$$k_e(\mathbf{x}, \mathbf{y}) = \phi\left(d_{\boldsymbol{\ell}}(\mathbf{x}, \mathbf{y})\right), \qquad d_{\boldsymbol{\ell}}(\mathbf{x}, \mathbf{y}) = \left(\sum_{i=1}^{d} \frac{(x_i - y_i)^2}{\ell_i^2} \right)^{1/2},$$

where the radial function $\phi : [0, \infty) \to \mathbb{R}$ and the length-scale parameters $\boldsymbol{\ell} \in (0, \infty)^d$ are to be specified.

Mátern Kernels The Mátern family of kernels is defined via the radial function

$$\phi_\nu(z) = \exp\left(-\sqrt{2s+1}\, z\right) \frac{s!}{(2s)!} \sum_{i=0}^{s} \frac{(s+i)!}{i!(s-i)!} \left(2\sqrt{2s+1}\, z\right)^{s-i}$$

for which $k_e \in C^{2s}(\mathcal{X} \times \mathcal{X})$ [Stein, 1999, Section 2.7]. The kernel k_e reproduces (up to an equivalent norm) the Sobolev space $H^{s+\frac{d+1}{2}}(\mathcal{X})$ [Wendland, 2004, Corollary 10.48]. The elements of $H^{s+\frac{d+1}{2}}(\mathcal{X})$ are functions whose mixed weak partial derivatives up to order $s+\frac{d+1}{2}$ exist as elements of $L^2(\mathcal{X})$, and so in particular $C^{s+\frac{d+1}{2}}(\mathcal{X}) \subset H^{s+\frac{d+1}{2}}(\mathcal{X})$. Conversely, from the Sobolev embedding theorem, $H^{s+\frac{d+1}{2}}(\mathcal{X}) \subset C^s(\mathcal{X})$. Thus $C^{s+\frac{d+1}{2}}(\mathcal{X}) \subset \mathcal{H}_{k_e}(\mathcal{X}) \subset C^s(\mathcal{X})$. It may be helpful to point out that, somewhat confusingly, in most of the statistical literature the Mátern kernel is defined in terms of a 'smoothness parameter' $\nu := s + \frac{1}{2}$ [Porcu et al., 2024]. From Section 2.4 of this paper onward, "Matérn ($s = m$)" refers to ϕ_ν with $\nu = m+\frac{1}{2}$. As an example, the Matérn-$\frac{5}{2}$ kernel that appears in Figure 1 would correspond to $s = 2$ in our framework.

Wendland Kernels Let $z_+^m := \max(0, z)^m$ and $(\mathcal{I}\varphi)(z) := \int_z^\infty t\varphi(t)\,\mathrm{d}t$. The Wendland family of kernels is defined via the radial function

$$\phi_{d,s}(z) = \mathcal{I}^s \varphi_{\lfloor \frac{d}{2} \rfloor + s + 1}(z), \qquad \varphi_m(z) = (1-z)_+^m$$

for $s \in \mathbb{N}_0$, for which $k_e \in C^{2s}(\mathcal{X} \times \mathcal{X})$; see Theorem 9.13 of Wendland [2004]. The kernel k_e reproduces (up to an equivalent norm) the Sobolev space $H^{s+\frac{d+1}{2}}(\mathcal{X})$, at least when $s \geq 1$ (for $s = 0$ we need $d \geq 3$); see Theorem 10.35 of Wendland [2004]. Thus again $C^{s+\frac{d+1}{2}}(\mathcal{X}) \subset \mathcal{H}_{k_e}(\mathcal{X}) \subset C^s(\mathcal{X})$. Wendland kernels are sometimes preferred to Matérn kernels due to their compact support [but they are not the only available alternative with compact support; Porcu et al., 2024].

Gaussian Kernels More generally, if the kernel k_e satisfies $k_e \in C^{2s}(\mathcal{X} \times \mathcal{X})$ with $\mathcal{X}$ an open subset of $\mathbb{R}^d$, then $\mathcal{H}_{k_e}(\mathcal{X}) \subset C^s(\mathcal{X})$; see Theorem 10.45 of Wendland [2004]. In particular the Gaussian kernel, defined by the radial function

$$\phi(z) = \exp\left(-z^2\right),$$

has continuous derivatives of all orders, so elements of the associated Hilbert space are $C^\infty(\mathcal{X})$.

B Proofs of Results in the Main Text

This appendix contains proofs for all theoretical results presented in the main text.

B.1 Sample Path Properties of Numerical Analysis-Informed GPs

This appendix presents sufficient conditions under which sample paths g from the numerical analysis-informed GP in (3) satisfy, with probability one, $g(\mathbf{x}) - g(\mathbf{0}) = O(b(\mathbf{x}))$ in the $\mathbf{x} \to \mathbf{0}$ limit. To be precise, we establish that there is a *version* g of $\mathcal{GP}(0, k)$ for which, with probability one, $g(\mathbf{x}) - g(\mathbf{0}) = O(b(\mathbf{x}))$. Recall that a stochastic process h is said to be a *version* of g if $h(\mathbf{x}) = g(\mathbf{x})$ with probability one, for all $\mathbf{x} \in \mathcal{X} \setminus \{\mathbf{0}\}$. This technical

consideration arises simply because stochastic processes can be altered on null sets, affecting their convergence properties while leaving their distribution unchanged.

Proposition 14 (Sample paths of numerical analysis-informed GPs)**.** *In the setting of Section 2.1, assume that k_e is Hölder continuous for some exponent; i.e. there exist $C, \alpha > 0$ such that $|k_e(\mathbf{x},\mathbf{x}) - k_e(\mathbf{x},\mathbf{x}')| \leq C\|\mathbf{x}-\mathbf{x}'\|^\alpha$ for all $\mathbf{x},\mathbf{x}' \in \mathcal{X}$, and that the set $\mathcal{X}$ is bounded. Then there is a version g of $\mathcal{GP}(0,k)$ for which, with probability one, $g(\mathbf{x}) - g(\mathbf{0}) = O(b(\mathbf{x}))$ in the $\mathbf{x} \to \mathbf{0}$ limit.*

Proof. Let $e(\mathbf{x}) := b(\mathbf{x})^{-1}(g(\mathbf{x}) - g(\mathbf{0}))$ for $\mathbf{x} \in \mathcal{X} \setminus \{\mathbf{0}\}$, which is well-defined since $b(\mathbf{x}) > 0$ for all $\mathbf{x} \in \mathcal{X} \setminus \{\mathbf{0}\}$. From (3), if $g \sim \mathcal{GP}(0,k)$ then the distribution of e is $\mathcal{GP}(0, k_e|_{\mathcal{X}\setminus\{\mathbf{0}\}})$, where $k_e|_{\mathcal{X}\setminus\{\mathbf{0}\}}(\mathbf{x},\mathbf{x}') = k_e(\mathbf{x},\mathbf{x}')$ for all $\mathbf{x},\mathbf{x}' \in \mathcal{X} \setminus \{\mathbf{0}\}$. Our task is equivalent to establishing that there is a version of e for which $e(\mathbf{x})$ is almost surely bounded as $\mathbf{x} \to \mathbf{0}$, but we will in fact establish the stronger result that there is a version of e for which $\mathbb{P}(\sup_{\mathbf{x}\in\mathcal{X}\setminus\{\mathbf{0}\}} |e(\mathbf{x})| < \infty) = 1$. From Markov's inequality, this follows if $\mathbb{E}[\sup_{\mathbf{x}\in\mathcal{X}\setminus\{\mathbf{0}\}} |e(\mathbf{x})|] < \infty$, and in fact it is sufficient to show that

$$\mathbb{E}\left[\sup_{\mathbf{x}\in\mathcal{X}\setminus\{\mathbf{0}\}} e(\mathbf{x})\right] < \infty. \tag{12}$$

Indeed, for any $\mathbf{x}_0 \in \mathcal{X} \setminus \{\mathbf{0}\}$, we have

$$\begin{aligned} |e(\mathbf{x})| &\leq |e(\mathbf{x}_0)| + |e(\mathbf{x}) - e(\mathbf{x}_0)| \\ &\leq |e(\mathbf{x}_0)| + \sup_{\mathbf{x}'\in\mathcal{X}\setminus\{\mathbf{0}\}} [e(\mathbf{x}') - e(\mathbf{x}_0)] - \inf_{\mathbf{x}'\in\mathcal{X}\setminus\{\mathbf{0}\}} [e(\mathbf{x}') - e(\mathbf{x}_0)] \end{aligned}$$

from which it follows that

$$\begin{aligned} \mathbb{E}\left[\sup_{\mathbf{x}\in\mathcal{X}\setminus\{\mathbf{0}\}} |e(\mathbf{x})|\right] &\leq \mathbb{E}[|e(\mathbf{x}_0)|] + \mathbb{E}\left[\sup_{\mathbf{x}'\in\mathcal{X}\setminus\{\mathbf{0}\}} e(\mathbf{x}') - e(\mathbf{x}_0)\right] - \mathbb{E}\left[\inf_{\mathbf{x}'\in\mathcal{X}\setminus\{\mathbf{0}\}} e(\mathbf{x}') - e(\mathbf{x}_0)\right] \\ &= \mathbb{E}[|e(\mathbf{x}_0)|] + 2\mathbb{E}\left[\sup_{\mathbf{x}'\in\mathcal{X}\setminus\{\mathbf{0}\}} e(\mathbf{x}')\right], \end{aligned}$$

where $\mathbb{E}[|e(\mathbf{x}_0)|] < \infty$ since $e(\mathbf{x}_0)$ is Gaussian, and where the final equality followed from $\mathbb{E}[e(\mathbf{x}_0)] = 0$ and symmetry of the GP.

Our main tools to establish (12) are entropy numbers and Dudley's theorem. The Hölder condition ensures that the induced pseudometric $\mathsf{d} : \mathcal{X} \times \mathcal{X} \to [0,\infty)$ defined via $\mathsf{d}(\mathbf{x},\mathbf{x}')^2 := k_e(\mathbf{x},\mathbf{x}) - 2k_e(\mathbf{x},\mathbf{x}') + k_e(\mathbf{x}',\mathbf{x}')$ satisfies $\mathsf{d}(\mathbf{x},\mathbf{x}') \leq \sqrt{2C}\|\mathbf{x}-\mathbf{x}'\|^{\alpha/2}$ for all $\mathbf{x},\mathbf{x}' \in \mathcal{X}$. Let $B_{\epsilon,\mathsf{d}}(\mathbf{x}) := \{\mathbf{x}' \in \mathcal{X} \setminus \{\mathbf{0}\} : \mathsf{d}(\mathbf{x},\mathbf{x}') < \epsilon\}$ denote an open d-ball of radius ϵ centred at $\mathbf{x}$, and let $N(\mathcal{X} \setminus \{\mathbf{0}\}, \mathsf{d}; \epsilon)$ denote the *entropy number*; the minimal number of open d-balls of radius ϵ required to cover $\mathcal{X} \setminus \{\mathbf{0}\}$. The boundedness condition on $\mathcal{X}$ ensures that the entropy number is well-defined. Dudley's theorem states that, in our context, there is a version e of $\mathcal{GP}(0,k_e)$ such that

$$\mathbb{E}\left[\sup_{\mathbf{x}\in\mathcal{X}\setminus\mathbf{0}} e(\mathbf{x})\right] \leq 24 \int_0^\infty \sqrt{\log N(\mathcal{X} \setminus \{\mathbf{0}\}, \mathsf{d}; \epsilon)}\, \mathrm{d}\epsilon; \tag{13}$$

see Theorem 11.17 in Ledoux and Talagrand [1991]. Our task is now to establish that the integral in (13) is finite, and to this end we make use of a simple upper bound on the entropy number for d in terms of the entropy number for the usual Euclidean distance $\mathsf{e}(\mathbf{x}, \mathbf{x}') := \|\mathbf{x} - \mathbf{x}'\|$ by noting that

$$\begin{aligned} B_{\epsilon,\mathsf{d}}(\mathbf{x}) &= \{\mathbf{x}' \in \mathcal{X} \setminus \{\mathbf{0}\} : \mathsf{d}(\mathbf{x}, \mathbf{x}') < \epsilon\} \\ &\supseteq \{\mathbf{x}' \in \mathcal{X} \setminus \{\mathbf{0}\} : \sqrt{2C}\|\mathbf{x} - \mathbf{x}'\|^{\alpha/2} < \epsilon\} = B_{(\epsilon/C)^{1/\alpha},\mathsf{e}}(\mathbf{x}), \end{aligned}$$

from which it follows that $N(\mathcal{X} \setminus \{\mathbf{0}\}, \mathsf{d}; \epsilon) \leq N(\mathcal{X} \setminus \{\mathbf{0}\}, \mathsf{e}; (\epsilon/C)^{1/\alpha})$. Since $\mathcal{X}$ is bounded, the entropy number for the Euclidean distance can be bounded by considering the number of Euclidean balls of radius $(\epsilon/C)^{1/\alpha}$ that are needed to cover a sufficiently large cube in $\mathbb{R}^d$, from which we obtain a bound of the form

$$N\left(\mathcal{X} \setminus \{\mathbf{0}\}, \mathsf{e}; \left(\frac{\epsilon}{C}\right)^{1/\alpha}\right) \leq \max\left\{1, \tilde{C}\left(\frac{C}{\epsilon}\right)^{d/\alpha}\right\}$$

for some constant $\tilde{C}$, from which the finiteness of the integral in (13) can be established. Indeed, letting $\epsilon_0 = \tilde{C}^{\alpha/d} C$, we have the bound

$$\begin{aligned} \int_0^\infty \sqrt{\log N(\mathcal{X}, \mathsf{d}; \epsilon)}\, \mathrm{d}\epsilon &\leq \int_0^\infty \sqrt{\max\left\{0, \frac{d}{\alpha}\log\left(\frac{\epsilon_0}{\epsilon}\right)\right\}}\, \mathrm{d}\epsilon \\ &= \sqrt{\frac{d}{\alpha}} \int_0^{\epsilon_0} \sqrt{\log\left(\frac{\epsilon_0}{\epsilon}\right)}\, \mathrm{d}\epsilon = \sqrt{\frac{\pi d}{\alpha}} \frac{\epsilon_0}{2} < \infty, \end{aligned}$$

as required. □

The Hölder and boundedness assumptions in Proposition 6 are weak and hold for all of the examples in this paper that we considered. The reader can find a similar result for dimension $d = 1$ and a polynomial error bound $b(x)$ stated without proof in Bect et al. [2021].

B.2 Derivation of the 'Objective' Prior Limit

This appendix contains standard calculations that can be found in references such as Karvonen et al. [2018], but we include them here to keep the paper self-contained. Let $f \sim \mathcal{GP}(0, k)$ where we fix finite values of $\sigma^2, k_0^2 > 0$ in the specification of k in (3). The distribution of f conditional upon the components $f(X_n)$ takes the familiar form

$$m_n[f](\mathbf{x}) = \mathbf{k}(\mathbf{x})^\top \mathbf{K}^{-1} f(X_n), \tag{14}$$

$$k_n[f](\mathbf{x}, \mathbf{x}') = k(\mathbf{x}, \mathbf{x}') - \mathbf{k}(\mathbf{x})^\top \mathbf{K}^{-1} \mathbf{k}(\mathbf{x}'); \tag{15}$$

see Chapter 2 of Rasmussen and Williams [2006]. Here $k(\mathbf{x}, \mathbf{x}') = \sigma^2\{k_0^2 + k_b(\mathbf{x}, \mathbf{x}')\}$, $\mathbf{k}(\mathbf{x}) = \sigma^2\{k_0^2 \mathbf{1} + \mathbf{k}_b(\mathbf{x})\}$, and $\mathbf{K} = \sigma^2\{k_0^2 \mathbf{1}\mathbf{1}^\top + \mathbf{K}_b\}$. Next we use the Woodbury matrix inversion identity to deduce that

$$\mathbf{K}^{-1} = \sigma^{-2}(k_0^2 \mathbf{1}\mathbf{1}^\top + \mathbf{K}_b)^{-1} = \sigma^{-2}\{\mathbf{K}_b^{-1} - \mathbf{K}_b^{-1}\mathbf{1}(k_0^{-2} + \mathbf{1}^\top \mathbf{K}_b^{-1}\mathbf{1})^{-1}\mathbf{1}^\top \mathbf{K}_b^{-1}\}. \tag{16}$$

Plugging this into (14) and (15), we obtain

$$m_n[f](\mathbf{x}) = \{k_0^2\mathbf{1} + \mathbf{k}_b(\mathbf{x})\}^\top\{\mathbf{K}_b^{-1} - \mathbf{K}_b^{-1}\mathbf{1}(k_0^{-2} + \mathbf{1}^\top\mathbf{K}_b^{-1}\mathbf{1})^{-1}\mathbf{1}^\top\mathbf{K}_b^{-1}\}f(X_n),$$
$$\frac{k_n[f](\mathbf{x},\mathbf{x}')}{\sigma^2} = k_0^2 + k_b(\mathbf{x},\mathbf{x}') - (k_0^2\mathbf{1} + \mathbf{k}_b(\mathbf{x}))^\top\left\{\mathbf{K}_b^{-1} - \frac{\mathbf{K}_b^{-1}\mathbf{1}\mathbf{1}^\top\mathbf{K}_b^{-1}}{(k_0^{-2} + \mathbf{1}^\top\mathbf{K}_b^{-1}\mathbf{1})}\right\}(k_0^2\mathbf{1} + \mathbf{k}_b(\mathbf{x}')). \tag{17}$$

Then, for small k_0^{-2}, we have from a Taylor expansion that

$$(k_0^{-2} + \mathbf{1}^\top\mathbf{K}_b^{-1}\mathbf{1})^{-1} = \frac{1}{\mathbf{1}^\top\mathbf{K}_b^{-1}\mathbf{1}} - \frac{k_0^{-2}}{(\mathbf{1}^\top\mathbf{K}_b^{-1}\mathbf{1})^2} + \frac{k_0^{-4}}{(\mathbf{1}^\top\mathbf{K}_b^{-1}\mathbf{1})^3} + O(k_0^{-6}) \tag{18}$$

so that

$$m_n[f](\mathbf{x}) = \frac{\mathbf{1}^\top\mathbf{K}_b^{-1}f(X_n)}{\mathbf{1}^\top\mathbf{K}_b^{-1}\mathbf{1}} + \mathbf{k}_b(\mathbf{x})^\top\mathbf{K}_b^{-1}\left\{f(X_n) - \left(\frac{\mathbf{1}^\top\mathbf{K}_b^{-1}f(X_n)}{\mathbf{1}^\top\mathbf{K}_b^{-1}\mathbf{1}}\right)\mathbf{1}\right\} + O(k_0^{-2}),$$
$$k_n[f](\mathbf{x},\mathbf{x}') = \sigma^2\left\{k_b(\mathbf{x},\mathbf{x}') - \mathbf{k}_b(\mathbf{x})^\top\mathbf{K}_b^{-1}\mathbf{k}_b(\mathbf{x}') + \frac{[\mathbf{k}_b(\mathbf{x})^\top\mathbf{K}_b^{-1}\mathbf{1} - 1][\mathbf{k}_b(\mathbf{x}')^\top\mathbf{K}_b^{-1}\mathbf{1} - 1]^\top}{\mathbf{1}^\top\mathbf{K}_b^{-1}\mathbf{1}}\right\}$$
$$+ O(k_0^{-2}),$$

which gives the stated limiting result.

B.3 Technical Results on Polynomial Reproduction

The proof that we present for Theorem 2 is based on local polynomial reproduction, and we aim for a result similar to Theorem 11.13 in Wendland [2004]. That result, however, aims for applicability to general domains and relies on an *interior cone condition* to ensure that fill distances based on balls can be meaningfully related to the approximation task. In our case, where the domain is axis-aligned, simpler and sharper results may be obtained by performing an alternative analysis based directly on boxes instead. Furthermore, we require a result for non-isotropic kernels, while Theorem 11.13 in Wendland [2004] assumes an isotropic kernel.

Our starting points is the following fundamental result on polynomial functions and scattered data:

Lemma 15 (Lemma 1 in Madych and Nelson [1992])**.** *Let* $\gamma_1 = 2$ *and* $\gamma_d = 2d(1+\gamma_{d-1})$ *for* $d > 1$*. Let* $\ell \in \mathbb{N}$ *with* $q \geq \gamma_d(\ell+1)$*. Let* $\mathcal{X} = [\mathbf{a}, \mathbf{a}+\lambda\mathbf{1}]$ *for some* $\mathbf{a} \in \mathbb{R}_+^d$ *and* $\lambda > 0$*. Divide* $\mathcal{X}$ *into* q^d *identical subcubes. If* $X_n \subset \mathcal{X}$ *is a set of* $n \geq q^d$ *points such that each subcube contains at least one of these points, then for all* $p \in \pi_\ell(\mathbb{R}^d)$*,*

$$\|p\|_{L^\infty(\mathcal{X})} \leq e^{2d\gamma_d(\ell+1)}\|p\|_{L^\infty(X_n)}.$$

Now let $\mathcal{X}$ be a domain and $X_n = \{\mathbf{x}_1, \dots, \mathbf{x}_n\} \subset \mathcal{X}$. The elementary tool that we use is the *sampling operator*

$$\begin{aligned} T_{X_n} : \pi_\ell(\mathcal{X}) &\to \mathbb{R}^n \\ p &\mapsto (p(\mathbf{x}_1), \dots, p(\mathbf{x}_n)) \end{aligned}$$

where $\pi_\ell(\mathcal{X})$ is equipped with the norm $\|\cdot\|_{L^\infty(\mathcal{X})}$. The set of pointwise evaluation functionals on X_n is called a *norming set* for $\pi_\ell(\mathcal{X})$ if the sampling operator T_{X_n} is injective into $\mathbb{R}^n$. For shorthand, we simply call such X_n a norming set. The terminology comes from the fact that such a T_{X_n} can be used to induce a norm on $\pi_\ell(\mathcal{X})$ using the norm on $\mathbb{R}^n$. For further details on sampling operators and norming sets, see Section 4 of Mhaskar et al. [2001]. For a continuous linear function $T : U \to V$ between normed spaces U and V, we let $\|T\|$ denote the operator norm $\sup_{u\neq 0} \|T(u)\|_V/\|u\|_U$. In what follows, Propositions 16 to 18 provide analogues of Theorems 3.4, 3.8, and 11.21 of Wendland [2004] that are adapted to the case of the box fill distance (as opposed to the ball fill distance) to obtain sharper constants for use in the present context.

Proposition 16 (Properties of the sampling operator). *Let $\mathcal{X} = [\mathbf{a}, \mathbf{a}+\lambda\mathbf{1}]$ for some $\mathbf{a} \in \mathbb{R}^d_+$ and $\lambda > 0$. Let $\ell \in \mathbb{N}$. Suppose that $\rho_{X_n,\mathcal{X}} \leq \lambda/(\gamma_d(\ell+1))$. Then X_n is a norming set and $\|T_{X_n}^{-1}\| \leq e^{2d\gamma_d(\ell+1)}$.*

Proof. If T_{X_n} is not injective then, since T_{X_n} is linear, there must be a $0 \neq p \in \pi_\ell(\mathcal{X})$ for which $T_{X_n}(p) = 0$. Let $0 \neq p \in \pi_\ell(\mathbb{R}^d)$. Our first task is to show that $T_{X_n}(p) \neq 0$. Since $\rho_{X_n,\mathcal{X}} \leq \lambda/(\gamma_d(\ell+1))$, the conditions of Lemma 15 are satisfied with $q = \gamma_d(\ell+1)$. Thus the conclusion of Lemma 15 holds, namely that

$$\|T_{X_n}(p)\|_\infty = \|p\|_{L^\infty(X_n)} \geq e^{-2d\gamma_d(\ell+1)}\|p\|_{L^\infty(\mathcal{X})} > 0.$$

Thus $T_{X_n}(p) \neq 0$, showing that T_{X_n} must be injective and X_n must be a norming set. Finally, we have also shown that

$$\|T_{X_n}^{-1}\| = \sup_{p\neq 0} \frac{\|p\|_{L^\infty(\mathcal{X})}}{\|T_{X_n}(p)\|} \leq \sup_{p\neq 0} \frac{\|p\|_{L^\infty(\mathcal{X})}}{\|T_{X_n}(p)\|_\infty} \leq e^{2d\gamma_d(\ell+1)},$$

as claimed. □

Using a norming set X_n, we can establish a global form of polynomial reproduction, as stated in the next result:

Proposition 17 (Global polynomial reproduction). *In the setting of Proposition 16, for each $\mathbf{x} \in \mathcal{X}$ there exists $\mathbf{u}(\mathbf{x}) \in \mathbb{R}^n$ such that*

- $p(\mathbf{x}) = \sum_{i=1}^n u_i(\mathbf{x})p(\mathbf{x}_i)$ *for all* $p \in \pi_\ell(\mathcal{X})$,
- $\sum_{i=1}^n |u_i(\mathbf{x})| \leq e^{2d\gamma_d(\ell+1)}$.

Proof. Define a function $f : \mathrm{range}(T_{X_n}) \to \mathbb{R}$ by $f(\mathbf{v}) = T_{X_n}^{-1}(\mathbf{v})(\mathbf{x})$. From the conclusion of Proposition 16 we have $\|f\| \leq \|T_{X_n}^{-1}\| \leq e^{2d\gamma_d(\ell+1)}$. By the Hahn–Banach theorem, f has a norm-preserving extension f_{ext} to $\mathbb{R}^n$. Since f_{ext} is a linear function on $\mathbb{R}^n$, it can be written as $f_{\mathrm{ext}}(\mathbf{v}) = \langle \mathbf{v}, \mathbf{u}\rangle$ from the Riesz representation theorem where $\|\mathbf{u}\| = \|f_{\mathrm{ext}}\| \leq e^{2d\gamma_d(\ell+1)}$. Then $p(\mathbf{x}) = f(T_{X_n}(p)) = f_{\mathrm{ext}}(T_{X_n}(p)) = \sum_{i=1}^n u_i p(\mathbf{x}_i)$, as required. □

The application of Proposition 17 to a collection of smaller hypercubes contained in $\mathcal{X}$ yields the following local form of polynomial reproduction that will be used to prove Theorem 2:

Proposition 18 (Local polynomial reproduction)**.** *Suppose that* $\mathcal{X} = [\mathbf{0}, \gamma \mathbf{1}]$*. Let* $\ell \in \mathbb{N}$ *and* $0 < \lambda \leq \gamma$*. Suppose that* $\rho_{X_n,\mathcal{X}} \leq \lambda/(\gamma_d(\ell+1))$*. Then for all* $\mathbf{x} \in \mathcal{X}$ *there exist numbers* $u_i(\mathbf{x})$ *with*

- $\sum_{i=1}^n u_i(\mathbf{x}) p(\mathbf{x}_i) = p(\mathbf{x})$ *for all* $p \in \pi_\ell(\mathcal{X})$*,*
- $\sum_{i=1}^n |u_i(\mathbf{x})| \leq e^{2d\gamma_d(\ell+1)}$ *,*
- $u_i(\mathbf{x}) = 0$ *if* $\|\mathbf{x} - \mathbf{x}_i\|_\infty > \lambda$ *.*

Proof. Given $\mathbf{x} \in \mathcal{X}$ we can find a box $\mathcal{X}_\mathbf{x} := [\mathbf{a}, \mathbf{a} + \lambda \mathbf{1}] \subset \mathcal{X}$ with $\mathbf{x} \in \mathcal{X}_\mathbf{x}$. Let $Y_n := X_n \cap \mathcal{X}_\mathbf{x} = \{\mathbf{y}_1, \ldots, \mathbf{y}_m\}$. The box $\mathcal{X}_\mathbf{x}$ and the set Y_n satisfy the conditions of Proposition 17 and thus there exists $\tilde{u}_i(\mathbf{x})$ such that

- $p(\mathbf{x}) = \sum_{i=1}^m \tilde{u}_i(\mathbf{x}) p(\mathbf{y}_i)$ for all $p \in \pi_\ell(\mathbb{R}^d)$,
- $\sum_{i=1}^m |\tilde{u}_i(\mathbf{x})| \leq e^{2d\gamma_d(\ell+1)}$.

From here we define $u_i(\mathbf{x}) = \tilde{u}_j(\mathbf{x})$ if $\mathbf{x}_i = \mathbf{y}_j$, otherwise we define $u_i(\mathbf{x}) = 0$, ensuring that $u_i(\mathbf{x}) = 0$ if $\|\mathbf{x} - \mathbf{x}_i\|_\infty > \lambda$. □

B.4 Proof of Theorem 2

This section contains the proof of Theorem 2. Before we begin, it is useful to recall that $\mathcal{H}_k(\mathcal{X})$ is endowed with the semi-norm $|f|_{\mathcal{H}_k(\mathcal{X})}$ which can be computed using either of the equivalent formulations $\|\mathbf{x} \mapsto f(\mathbf{x}) - f(\mathbf{0})\|_{\mathcal{H}_{k_b}(\mathcal{X})}$ or $\|\mathbf{x} \mapsto (f(\mathbf{x}) - f(\mathbf{0}))/b(\mathbf{x})\|_{\mathcal{H}_{k_e}(\mathcal{X})}$ [Paulsen and Raghupathi, 2016, Theorem 5.16]; here the latter formulation will be used. Let $\mathcal{S}(X_n)$ denote the set of linear functionals of the form $\mathsf{s}[f] = \mathbf{w}^\top f(X_n)$ whose weights are normalised such that $\mathbf{1}^\top \mathbf{w} = 1$.

Proposition 19. *The extrapolated mean estimator* $\mathsf{s}[f] = m_n[f](\mathbf{0})$ *from* (6) *minimises the worst case error*

$$\text{wce}(\mathsf{s}) := \sup \left\{ |f(\mathbf{0}) - \mathsf{s}[f]| \ : \ |f|_{\mathcal{H}_k(\mathcal{X})} \leq 1 \right\}$$

among all estimators $\mathsf{s} \in \mathcal{S}(X_n)$*.*

Proof. Consider a general element $\mathsf{s} \in \mathcal{S}(X_n)$, which has the form $\mathsf{s}[f] = \mathbf{w}^\top f(X_n)$ where $\mathbf{1}^\top \mathbf{w} = 1$. Since the weights $\mathbf{w}$ are normalised, s is exact on constant functions, and thus $f(\mathbf{0}) - \mathsf{s}[f] = \mathsf{s}[f(\mathbf{0}) - f]$ where $f(\mathbf{0}) - f \in \mathcal{H}_{k_b}(\mathcal{X})$. It follows that

$$\text{wce}(\mathsf{s}) = \sup \left\{ |\mathsf{s}[f]| \ : \ \|f\|_{\mathcal{H}_{k_b}(\mathcal{X})} \leq 1 \right\}.$$

It has been assumed that the elements of X_n are distinct. The Riesz representer of the error functional $f \mapsto f(\mathbf{0}) - \mathbf{w}^\top f(X_n)$ is $k_b(\mathbf{0}, \cdot) - \mathbf{w}^\top \mathbf{k}_b(\cdot)$ and, since $\mathrm{wce}(\mathsf{s})$ is the operator norm of the error functional and Hilbert spaces are self-dual, we have that

$$\begin{aligned} \mathrm{wce}(\mathsf{s})^2 = \left\| k_b(\mathbf{0}, \cdot) - \mathbf{w}^\top \mathbf{k}_b(\cdot) \right\|^2_{\mathcal{H}_{k_b}(\mathcal{X})} &= k_b(\mathbf{0}, \mathbf{0}) - 2\mathbf{w}^\top \mathbf{k}_b(\mathbf{0}) + \mathbf{w}^\top \mathbf{K}_b \mathbf{w} \\ &= \mathbf{w}^\top \mathbf{K}_b \mathbf{w} \end{aligned} \tag{19}$$

where the final equality follows since $k_b(\mathbf{0}, \mathbf{x}) = b(\mathbf{0}) b(\mathbf{x}) k_e(\mathbf{0}, \mathbf{x}) = 0$ for all $\mathbf{x} \in \mathcal{X}$. This leaves a quadratic form in $\mathbf{w}$ and we seek the minimum subject to $\mathbf{1}^\top \mathbf{w} = 1$. If we consider the Lagrangian

$$\mathcal{L}(\mathbf{w}, \lambda) = \mathbf{w}^\top \mathbf{K}_b \mathbf{w} + \lambda(\mathbf{1}^\top \mathbf{w} - 1)$$

then we have a critical point when $\partial_{\mathbf{w}} \mathcal{L} = 2\mathbf{K}_b \mathbf{w} + \lambda \mathbf{1} = 0$, so that $\mathbf{w} = -\frac{\lambda}{2} \mathbf{K}_b^{-1} \mathbf{1}$. Enforcing the normalisation constraint leads to $\mathbf{w} = \mathbf{K}_b^{-1} \mathbf{1} / (\mathbf{1}^\top \mathbf{K}_b^{-1} \mathbf{1})$, which means that $\mathsf{s}[f] = \mathbf{w}^\top f(X_n) = m_n[f](\mathbf{0})$, as claimed. □

The proof of Theorem 2 below employs the *multi-index* notation, meaning that for $\boldsymbol{\beta} \in \mathbb{N}_0^d$ we let $|\boldsymbol{\beta}| := \beta_1 + \cdots + \beta_d$, $\boldsymbol{\beta}! := \beta_1 \cdots \beta_d$, and $\mathbf{z}^{\boldsymbol{\beta}} := z_1^{\beta_1} \cdots z_d^{\beta_d}$ for $\mathbf{z} \in \mathbb{R}^d$.

Proof of Theorem 2. Assume without loss of generality that $|f|_{\mathcal{H}_k(\mathcal{X})} \neq 0$, since otherwise $m_n^h[f] = 0$ is trivially exact. Let $\mathbf{x}_i^h = h\mathbf{x}_i$ and $k_b(\mathbf{x}, \mathbf{y}) = b(\mathbf{x}) b(\mathbf{y}) k_e(\mathbf{x}, \mathbf{y})$ in shorthand. Introduce the positive semi-definite quadratic form

$$Q(\mathbf{u}) = \sum_{i=1}^n \sum_{j=1}^n u_i u_j k_b(\mathbf{x}_i^h, \mathbf{x}_j^h) \tag{20}$$

and note from Proposition 19 that

$$\frac{|f(\mathbf{0}) - m_n^h[f](\mathbf{0})|}{|f|_{\mathcal{H}(k)}} \leq \mathrm{wce}(m_n^h[f]) = \min \left\{ Q(\mathbf{u})^{1/2} : \mathbf{u} \in \mathbb{R}^n, \ \sum_{i=1}^n u_i = 1 \right\}. \tag{21}$$

Thus we can bound the relative error by the square root of $Q(\mathbf{u})$ for any choice of $\mathbf{u} \in \mathbb{R}^n$ for which $\sum_{i=1}^n u_i = 1$. Our choice of $\mathbf{u}$ will be based on polynomial reproduction, as described next.

From Taylor's theorem, since $k_e(\mathbf{x}, \cdot) \in C^{2s}(\mathcal{X}_h)$,

$$k_e(\mathbf{x}, \mathbf{y}) = \sum_{|\boldsymbol{\beta}| < 2s} \frac{\partial_{\mathbf{z}}^{\boldsymbol{\beta}} k_e(\mathbf{x}, \mathbf{z})|_{\mathbf{z} = \mathbf{x}}}{\boldsymbol{\beta}!} (\mathbf{y} - \mathbf{x})^{\boldsymbol{\beta}} + R(\mathbf{x}, \mathbf{y}), \qquad |R(\mathbf{x}, \mathbf{y})| \leq C_{\mathbf{x}}^{(2s)} \|\mathbf{x} - \mathbf{y}\|^{2s} \tag{22}$$

for all $\mathbf{x}, \mathbf{y} \in \mathcal{X}_h$, where the constants $C_{\mathbf{x}}^{(2s)} := ((2s)!)^{-1} \sup_{\mathbf{z} \in \mathcal{X}_h} \sum_{|\boldsymbol{\beta}| = 2s} \partial_{\mathbf{z}}^{\boldsymbol{\beta}} k_e(\mathbf{x}, \mathbf{z})$ are uniformly bounded by $C_k^{(2s)} := \sup_{\mathbf{x} \in \mathcal{X}} C_{\mathbf{x}}^{(2s)}$ since $\mathcal{X}$ is compact. Plugging this into (20) gives that

$$Q(\mathbf{u}) = \sum_{i=1}^n u_i b(\mathbf{x}_i^h) \sum_{j=1}^n u_j b(\mathbf{x}_j^h) \left(\sum_{|\boldsymbol{\beta}| < 2s} \frac{\partial_{\mathbf{z}}^{\boldsymbol{\beta}} k_e(\mathbf{x}_i^h, \mathbf{z})|_{\mathbf{z} = \mathbf{x}_i^h}}{\boldsymbol{\beta}!} (\mathbf{x}_j^h - \mathbf{x}_i^h)^{\boldsymbol{\beta}} + R(\mathbf{x}_i^h, \mathbf{x}_j^h) \right).$$

Since $b(\mathbf{x}_j^h)(\mathbf{x}_j^h - \mathbf{x}_i^h)^{\boldsymbol{\beta}}$ is a polynomial in $\mathbf{x}_j^h$ of total degree at most $r + |\boldsymbol{\beta}|$ and $|\boldsymbol{\beta}| < 2s$, we aim to pick a vector $\mathbf{u}$ for which local (at $\mathbf{0}$) polynomial reproduction on X_n^h occurs up to polynomials of total order $\ell := r + (2s-1)$. Set $\lambda := \gamma_d(\ell+1)\rho_{X_n^h,\mathcal{X}_h} = \gamma_d(\ell+1)h\rho_{X_n,\mathcal{X}}$, so that our assumption on the box fill distance implies $0 < \lambda \le h$, and trivially $\rho_{X_n^h,\mathcal{X}_h} \le \lambda/(\gamma_d(\ell+1))$. Thus the conditions of Proposition 18 are satisfied, and there exists $\mathbf{u} \in \mathbb{R}^n$ such that

- $\sum_{i=1}^n u_i p(\mathbf{x}_i^h) = p(\mathbf{0})$ for all $p \in \pi_\ell(\mathcal{X}_h)$,
- $\sum_{i=1}^n |u_i| \le e^{2d\gamma_d(\ell+1)}$,
- $u_i = 0$ if $\mathbf{x}_i^h \notin [\mathbf{0}, \lambda\mathbf{1}]$.

For this choice of $\mathbf{u}$ it follows from the local polynomial reproduction property that, for $|\boldsymbol{\beta}| < 2s$,

$$\sum_{j=1}^n u_j b(\mathbf{x}_j^h)(\mathbf{x}_j^h - \mathbf{x}_i^h)^{\boldsymbol{\beta}} = b(\mathbf{x})(\mathbf{x} - \mathbf{x}_i^h)^{\boldsymbol{\beta}}\big|_{\mathbf{x}=\mathbf{0}} = b(\mathbf{0})(-\mathbf{x}_i^h)^{\boldsymbol{\beta}} = \mathbf{0}$$

since $b(\mathbf{0}) = 0$, and thus, recalling that $u_i = 0$ whenever $\mathbf{x}_i^h \notin [\mathbf{0}, \lambda\mathbf{1}]$,

$$Q(\mathbf{u}) = \sum_{i=1}^n u_i b(\mathbf{x}_i^h) \sum_{j=1}^n u_j b(\mathbf{x}_j^h) R(\mathbf{x}_i^h, \mathbf{x}_j^h) \le \|\mathbf{u}\|_1^2 \|b\|_{L^\infty(\mathcal{X}_h)}^2 \sup_{\mathbf{x},\mathbf{y} \in [\mathbf{0},\gamma\mathbf{1}]} |R(\mathbf{x}, \mathbf{y})|,$$

where by construction $\|\mathbf{u}\|_1 \le e^{2d\gamma_d(\ell+1)}$. The final term can be bounded using the error estimate in (22):

$$\begin{aligned} \sup_{\mathbf{x},\mathbf{y} \in [\mathbf{0},\gamma\mathbf{1}]} |R(\mathbf{x}, \mathbf{y})| \le \sup_{\mathbf{x} \in [\mathbf{0},\gamma\mathbf{1}]} C_{\mathbf{x}}^{(2s)} \times \|\mathbf{0} - \lambda\mathbf{1}\|^{2s} &\le C_k^{(2s)} \frac{d^s}{(2s)!} \lambda^{2s} \\ &= C_k^{(2s)} \frac{d^s}{(2s)!} (\gamma_d(\ell+1)h\rho_{X_n,\mathcal{X}})^{2s}, \end{aligned}$$

leading to the claimed overall bound $\mathrm{wce}(m_n^h[f]) \le C_{r,s} h^s \rho_{X_n,\mathcal{X}}^s \|b\|_{L^\infty(\mathcal{X}_h)}$ on the relative error, where $C_{r,s} := (C_k^{(2s)})^{1/2} d^{s/2} e^{2d\gamma_d(\ell+1)} \gamma_d^s (\ell+1)^s / \sqrt{(2s)!}$. □

B.5 Proof of Theorem 4

Proof of Theorem 4. The assumption on the growth of the derivatives of k_e implies that $C_k^{(2s)} \le C_k^{2s}$ for the assumed constant C_k, and we employ this bound throughout. Now, to more easily track the s-dependent constants in the bound of Theorem 2 we employ a simpler upper bound

$$C_{r,s} h^s \rho_{X_n,\mathcal{X}}^s = \frac{C_k^s d^{s/2} e^{2d\gamma_d(r+2s)} \gamma_d^s (r+2s)^s}{\sqrt{(2s)!}} h^s \rho_{X_n,\mathcal{X}}^s \le e^{2d\gamma_d r} \left(\frac{C_k d^{1/2} e^{4d\gamma_d + 1} h}{2s} \right)^s \tag{23}$$

which holds whenever $\rho_{X_n,\mathcal{X}} \leq 1/(\gamma_d(r+2s))$, and where we used the elementary fact $1/\sqrt{(2s)!} \leq (e/(2s))^s$ to obtain this simpler bound.

The idea of this proof is to pick a particular value of $s \in \mathbb{N}_0$ that will be $\rho_{X_n,\mathcal{X}}$-dependent. For (23) to hold we require $\rho_{X_n,\mathcal{X}} \leq 1/(\gamma_d(r+2s))$, and the largest such s for which this requirement is satisfied is

$$s^\star := \left\lfloor -\frac{r}{2} + \frac{1}{2\gamma_d \rho_{X_n,\mathcal{X}}} \right\rfloor. \tag{24}$$

The assumption $\rho_{X_n,\mathcal{X}} \leq 1/(2\gamma_d(r+1))$ implies $s^\star \geq 1/(4\gamma_d\rho_{X_n,\mathcal{X}}) \geq 0$, so s is well-defined as an element of $\mathbb{N}_0$. Further, the assumption $\rho_{X_n,\mathcal{X}} \leq 1/(2d^{1/2}\gamma_d e^{4d\gamma_d+1})$ implies $t \mapsto [(\frac{1}{2}d^{1/2}e^{4d\gamma_d+1}h)/t]^t$ is decreasing on $t \in [1/(4\gamma_d\rho_{X_n,\mathcal{X}}), \infty)$ for any $h \in (0,1]$. Thus we can replace s by $1/(4\gamma_d\rho_{X_n,\mathcal{X}})$ in (23) to obtain the upper bound

$$C_{r,s}h^s\rho_{X_n,\mathcal{X}}^s \leq e^{2d\gamma_d r}\left(\frac{\frac{1}{2}C_k d^{1/2}e^{4d\gamma_d+1}h}{1/(4\gamma_d\rho_{X_n,\mathcal{X}})}\right)^{1/(4\gamma_d\rho_{X_n,\mathcal{X}})} = C_{n,r,s}h^{\frac{1}{4\gamma_d\rho_{X_n,\mathcal{X}}}}$$

where $C_{n,r,s} := (2C_k d^{1/2}\gamma_d e^{4d\gamma_d+1}\rho_{X_n,\mathcal{X}})^{1/(4\gamma_d\rho_{X_n,\mathcal{X}})}$ is a h-independent constant. The assumptions we made above on the box fill distance are satisfied when $\rho_{X_n,\mathcal{X}} \leq \min\{1/(2\gamma_d(r+1)), 1/(2d^{1/2}\gamma_d e^{4d\gamma_d+1})\}$. □

B.6 Verifying the Assumptions for Example 5

The aim of this appendix is to verify that the function $\psi(x) = \sin(10x) + 1_{x>0}x^{s+4}$ satisfies $\psi(x) = c_0 + c_1x + c_2x^2 + c_3(x)x^3$ for some $c_0, c_1, c_2 \in \mathbb{R}$ and $c_3 \in H^{s+1}(\mathcal{O})$ where $\mathcal{O} = (-\delta, \delta)$, $\delta > 0$, is an open neighbourhood of 0 and we recall that $H^{s+1}(\mathcal{O})$ is the Sobolev space of $s+1$ times weakly differentiable functions on $\mathcal{O}$; see Appendix A. It then follows from Appendix A that $x \mapsto c_3(x)$ and $x \mapsto c_3(-x)$ are elements of $\mathcal{H}_{k_e}(\mathcal{X})$ whenever k_e is either the Mátern or Wendland kernel with smoothness s and $\mathcal{X} = [0, \delta)$, since these kernels reproduce (up to an equivalent norm) $H^{s+1}(\mathcal{X})$ in dimension $d = 1$.

From linearity, it suffices to establish this fact separately for $\psi_1(x) := \sin(10x)$ and $\psi_2(x) := 1_{x>0}x^{s+4}$. For the first term, since the trigonometric functions are real-analytic, we have a convergent power series $\psi_1(x) = \sum_{i=0}^\infty \tilde{c}_i x^i$ for $x \in [-\epsilon, \epsilon]$ for some $\epsilon > 0$. Thus $\psi_1(x) = \tilde{c}_0 + \tilde{c}_1 x + \tilde{c}_2 x^2 + c_3(x)x^3$ with $c_3(x) = \sum_{i=3}^\infty \tilde{c}_i x^{i-3}$, and our task is to show that this latter series is convergent in a neighbourhood of $x = 0$. To this end, we restrict attention to $x \in [-\epsilon/2, \epsilon/2]$, and then use the ratio test

$$\frac{|\tilde{c}_i x^{i-3}|}{|\tilde{c}_i \epsilon^i|} = \frac{1}{|x|^3}\left|\frac{x}{\epsilon}\right|^i \leq \frac{1}{|x|^3}\frac{1}{2^i} \to 0 \quad \text{as} \quad i \to \infty$$

to deduce absolute convergence of the series, as required. For the second term, we take $c_0 = c_1 = c_2 = 0$, so that $\psi_2(x) = c_3(x)x^3$ where $c_3(x) = 1_{x>0}x^{s+1}$, observing that c_3 has an $(s+1)$-order weak derivative $c_3^{(s+1)}(x)$ taking the value 0 on $x \leq 0$ and $(s+1)!$ on $x > 0$, so that $c_3 \in H^{s+1}(\mathcal{O})$.

B.7 Proof of Proposition 6 and Corollary 7

Proof of Proposition 6. Our assumption implies $(e_n)_{n\in\mathbb{N}}$ is Cauchy and thus this sequence converges to a limit, denoted $e_\infty \in \mathbb{R}$. Let $\varphi : [0,\infty) \to [0,1]$ be a smooth function with $\varphi(0) = 0$, $\varphi = 1$ on $[1,\infty)$, and derivatives uniformly bounded. Let

$$e(\mathbf{x}) := \begin{cases} e_\infty & \text{if} \quad \mathbf{x} = \mathbf{0}, \\ e_{n+1} + (e_n - e_{n+1}) \prod_{i=1}^d \varphi\left(\frac{x_i - \mathbf{x}_{n+1,i}}{\mathbf{x}_{n,i} - \mathbf{x}_{n+1,i}}\right) & \text{if} \quad \mathbf{x} \in [\mathbf{0}, \mathbf{x}_n] \setminus [\mathbf{0}, \mathbf{x}_n], \\ e_1 & \text{if} \quad \mathbf{x} \notin [\mathbf{0}, \mathbf{x}_1] \end{cases}$$

so that $e(\mathbf{x}_n) = e_n$ and $e(\mathbf{0}) = e_\infty$. Now set $f(\mathbf{x}) = y_\infty + b(\mathbf{x})e(\mathbf{x})$. To establish $|f|_{\mathcal{H}_k(\mathcal{X})} < \infty$ it suffices to establish that $\lim_{\mathbf{x}\to\mathbf{0}} \partial^{\boldsymbol{\beta}} e(\mathbf{x})$ exists and is finite for all $|\boldsymbol{\beta}| = p$, since then we will have $e \in C^p(\mathcal{X}) \subset \mathcal{H}(k_e)$. This is indeed the case, since on $[\mathbf{0}, \mathbf{x}_n] \setminus [\mathbf{0}, \mathbf{x}_n]$ we have that

$$\partial^{\boldsymbol{\beta}} e(\mathbf{x}) = (e_n - e_{n+1}) \prod_{i=1}^d \varphi^{(\beta_i)} \left(\frac{x_i - \mathbf{x}_{n+1,i}}{\mathbf{x}_{n,i} - \mathbf{x}_{n+1,i}}\right) \frac{1}{(\mathbf{x}_{n,i} - \mathbf{x}_{n+1,i})^{\beta_i}}.$$

where the $\varphi^{(\beta_i)}$ terms are uniformly bounded and the remaining terms vanish since

$$|e_n - e_{n+1}| \prod_{i=1}^d \frac{1}{(\mathbf{x}_{n,i} - \mathbf{x}_{n+1,i})^{\beta_i}} \leq \frac{|e_n - e_{n+1}|}{\min(\mathbf{x}_n - \mathbf{x}_{n+1})^p}$$

where $p = \beta_1 + \cdots + \beta_d$, and this final bound was assumed to vanish. □

Proof of Corollary 7. The renormalised error is $e_n = C_1 + C_2 x_n^{p+1} + O(x_n^{p+2})$, so that

$$\frac{e_n - e_{n+1}}{(x_n - x_{n+1})^p} = C_2 \frac{(x_n^{p+1} - x_{n+1}^{p+1})}{(x_n - x_{n+1})^p} + \frac{O(x_n^{p+2}) + O(x_{n+1}^{p+2})}{(x_n - x_{n+1})^p} \to 0.$$

Our assumptions on the sequence $(x_n)_{n\in\mathbb{N}}$ imply that both terms vanish in the $n \to \infty$ limit. □

B.8 Proof of Proposition 9

First we establish the correctness of the algebraic identity (8) given in the main text. Again, it is useful to recall that $\mathcal{H}_k(\mathcal{X})$ is endowed with the semi-norm $|f|_{\mathcal{H}_k(\mathcal{X})}$ which can be computed using either of the equivalent formulations $\|\mathbf{x} \mapsto f(\mathbf{x}) - f(\mathbf{0})\|_{\mathcal{H}_{k_b}(\mathcal{X})}$ or $\|\mathbf{x} \mapsto (f(\mathbf{x}) - f(\mathbf{0}))/b(\mathbf{x})\|_{\mathcal{H}_{k_e}(\mathcal{X})}$ [Paulsen and Raghupathi, 2016, Theorem 5.16]; here the latter formulation will be used.

Proposition 20. *The estimator* $\sigma_n^2[f] := \frac{1}{n}|m_n[f]|^2_{\mathcal{H}_k(\mathcal{X})}$ *has the explicit form*

$$\sigma_n^2[f] = \frac{1}{n}\left[f(X_n)^\top \mathbf{K}_b^{-1} f(X_n) - \frac{(\mathbf{1}^\top \mathbf{K}_b^{-1} f(X_n))^2}{\mathbf{1}^\top \mathbf{K}_b^{-1} \mathbf{1}}\right]$$

given in (8) *of the main text.*

Proof. Let $\bar{m}_n[f] = m_n[f] - m_n[f](\mathbf{0})$. From direct calculation,

$$\begin{aligned}
|m_n[f]|^2_{\mathcal{H}_k(\mathcal{X})} &= \|\bar{m}_n[f]\|^2_{\mathcal{H}_{k_b}(\mathcal{X})} \\
&= \left\| \mathbf{k}_b(\cdot)^\top \mathbf{K}_b^{-1} \left\{ f(X_n) - \left(\frac{\mathbf{1}^\top \mathbf{K}_b^{-1} f(X_n)}{\mathbf{1}^\top \mathbf{K}_b^{-1} \mathbf{1}} \right) \mathbf{1} \right\} \right\|^2_{\mathcal{H}_{k_b}(\mathcal{X})} \\
&= \left\{ f(X_n) - \left(\frac{\mathbf{1}^\top \mathbf{K}_b^{-1} f(X_n)}{\mathbf{1}^\top \mathbf{K}_b^{-1} \mathbf{1}} \right) \mathbf{1} \right\}^\top \mathbf{K}_b^{-1} \left\{ f(X_n) - \left(\frac{\mathbf{1}^\top \mathbf{K}_b^{-1} f(X_n)}{\mathbf{1}^\top \mathbf{K}_b^{-1} \mathbf{1}} \right) \mathbf{1} \right\} \\
&= f(X_n)^\top \mathbf{K}_b^{-1} f(X_n) - \frac{(\mathbf{1}^\top \mathbf{K}_b^{-1} f(X_n))^2}{\mathbf{1}^\top \mathbf{K}_b^{-1} \mathbf{1}},
\end{aligned}$$

which completes the proof. □

Next we present a general result, which forms the crux of the argument in our proof of Proposition 9:

Proposition 21. *Assume that $f \in \mathcal{H}_k(\mathcal{X})$. Then*

$$\frac{|f(\mathbf{0}) - m_n[f](\mathbf{0})|}{\sqrt{k_n[f](\mathbf{0},\mathbf{0})}} \leq \frac{|f|_{\mathcal{H}_k(\mathcal{X})}}{\sigma_n[f]}.$$

Proof. From (19) in the the proof of Proposition 19, we have $\mathrm{wce}(\mathsf{s})^2 = \mathbf{w}^\top \mathbf{K}_b \mathbf{w}$ for any algorithm $\mathsf{s}[f] = \mathbf{w}^\top f(X_n)$ with $\mathbf{1}^\top \mathbf{w} = 1$. In particular, we can consider the algorithm $s[f] = m_n[f](\mathbf{0})$, which has $\mathbf{w} = \mathbf{K}_b^{-1}\mathbf{1}/(\mathbf{1}^\top \mathbf{K}_n^{-1}\mathbf{1})$, to see that

$$\mathrm{wce}(\mathsf{s})^2 = \left(\frac{\mathbf{K}_b^{-1}\mathbf{1}}{\mathbf{1}^\top \mathbf{K}_b^{-1}\mathbf{1}} \right)^\top \mathbf{K}_b \left(\frac{\mathbf{K}_b^{-1}\mathbf{1}}{\mathbf{1}^\top \mathbf{K}_b^{-1}\mathbf{1}} \right) = \frac{1}{\mathbf{1}^\top \mathbf{K}_b^{-1}\mathbf{1}} = \frac{k_n[f](\mathbf{0},\mathbf{0})}{\sigma_n^2[f]}.$$

This implies that

$$\frac{|f(\mathbf{0}) - m_n[f](\mathbf{0})|}{\sqrt{k_n[f](\mathbf{0},\mathbf{0})}} \leq \frac{\mathrm{wce}(\mathsf{s})|f|_{\mathcal{H}_k(\mathcal{X})}}{\sqrt{k_n[f](\mathbf{0},\mathbf{0})}} \leq \frac{|f|_{\mathcal{H}_k(\mathcal{X})}}{\sigma_n[f]},$$

from which the result is established. □

Proof of Proposition 9. For $f \in \mathcal{H}_k(\mathcal{X})$ we have the decomposition $f(\mathbf{x}) = f(\mathbf{0}) + \bar{f}(\mathbf{x})$ with $\bar{f} \in \mathcal{H}_{k_b}(\mathcal{X})$, and $|f|_{\mathcal{H}_k(\mathcal{X})} = \|\bar{f}\|_{\mathcal{H}_{k_b}(\mathcal{X})}$. From Proposition 21 and $\sigma_n^2[f] = \frac{1}{n}|m_n[f]|^2_{\mathcal{H}_k(\mathcal{X})}$, it follows that

$$\frac{|f(\mathbf{0}) - m_n^h[f](\mathbf{0})|}{\sqrt{k_n^h[f](\mathbf{0},\mathbf{0})}} \leq \frac{n|f|_{\mathcal{H}_k(\mathcal{X})}}{|m_n^h[f]|_{\mathcal{H}_k(\mathcal{X})}} = \frac{n\|\bar{f}\|_{\mathcal{H}_{k_b}(\mathcal{X})}}{\|\bar{m}_n^h[f]\|_{\mathcal{H}_{k_b}(\mathcal{X})}},$$

where similarly we have decomposed $m_n^h[f](\mathbf{x}) = m_n^h[f](\mathbf{0}) + \bar{m}_n^h[f](\mathbf{x})$. Since $\bar{f}$ and $\bar{m}_n^h[f]$ are elements of $\mathcal{H}_{k_b}(\mathcal{X})$ and $k_b(\mathbf{x},\mathbf{x}') = b(\mathbf{x})b(\mathbf{x}')k_e(\mathbf{x},\mathbf{x}')$, we may write

$$\bar{f}(\mathbf{x}) = b(\mathbf{x})e(\mathbf{x}) \quad \text{and} \quad \bar{m}_n^h[f](\mathbf{x}) = b(\mathbf{x})\tilde{e}_h(\mathbf{x})$$

for certain $e, \tilde{e}_h \in \mathcal{H}_{k_e}(\mathcal{X})$, with $\|\bar{f}\|_{\mathcal{H}_{k_b}(\mathcal{X})} = \|e\|_{\mathcal{H}_{k_e}(\mathcal{X})}$ and $\|\bar{m}_n^h[f]\|_{\mathcal{H}_{k_b}(\mathcal{X})} = \|\tilde{e}_h\|_{\mathcal{H}_{k_e}(\mathcal{X})}$. Therefore

$$\frac{|f(\mathbf{0}) - m_n^h[f](\mathbf{0})|}{\sqrt{k_n^h[f](\mathbf{0},\mathbf{0})}} \leq \frac{n\|e\|_{\mathcal{H}_{k_e}(\mathcal{X})}}{\|\tilde{e}_h\|_{\mathcal{H}_{k_e}(\mathcal{X})}}. \tag{25}$$

By construction, $m_n^h[f](h\mathbf{x}_i) = f(h\mathbf{x}_i)$ for $i = 1, \ldots, n$. Because $f(\mathbf{x}) = f(\mathbf{0}) + b(\mathbf{x})e(\mathbf{x})$, the function $\tilde{e}_h$ satisfies

$$\tilde{e}_h(h\mathbf{x}_i) = e(h\mathbf{x}_i) + b(h\mathbf{x}_i)^{-1}[f(\mathbf{0}) - m_n^h[f](\mathbf{0})]$$

for each $i = 1, \ldots, n$. The assumptions of Theorem 2 with $s = 1$ are satisfied, and thus

$$b(h\mathbf{x}_i)^{-1}|f(\mathbf{0}) - m_n^h[f](\mathbf{0})| \leq C_{r,1}h\rho_{X_n,\mathcal{X}}\frac{\|b\|_{L^\infty(\mathcal{X}_h)}}{b(h\mathbf{x}_i)}|f|_{\mathcal{H}_k(\mathcal{X})} \to 0$$

as $h \to 0$ since, b being a polynomial, $\lim_{h\to 0}\|b\|_{L^\infty(\mathcal{X}_h)}b(h\mathbf{x}_i)^{-1} < \infty$. The continuity of e, which follows from $e \in \mathcal{H}_{k_e}(\mathcal{X})$ and the continuity of k_e, then implies that $\tilde{e}_h(h\mathbf{x}_i) \to e(\mathbf{0})$ as $h \to 0$. Let $c \in \mathbb{R}$. The function $g(\mathbf{x}) := c\, k_e(\mathbf{x}, \mathbf{x}')k_e(\mathbf{x}', \mathbf{x}')^{-1}$ has minimal norm among all functions in $\mathcal{H}_{k_e}(\mathcal{X})$ that equal c at $\mathbf{x}'$ [Paulsen and Raghupathi, 2016, Corollary 3.5]. By the reproducing property $\langle k_e(\cdot, \mathbf{x}_1), k_e(\cdot, \mathbf{x}_2)\rangle_{\mathcal{H}_{k_e}(\mathcal{X})} = k_e(\mathbf{x}_1, \mathbf{x}_2)$ in the RKHS inner product, from which it follows that the norm of this function is

$$\|g\|_{\mathcal{H}_{k_e}(\mathcal{X})} = \langle g, g\rangle^{1/2}_{\mathcal{H}_{k_e}(\mathcal{X})} = |c|\, k_e(\mathbf{x}', \mathbf{x}')^{1/2}.$$

Consequently, setting $\mathbf{x}' = h\mathbf{x}_1$ and $c = \tilde{e}_h(h\mathbf{x}_1)$ gives

$$\|\tilde{e}_h\|_{\mathcal{H}_{k_e}(\mathcal{X})} \geq |\tilde{e}_h(h\mathbf{x}_1)|k_e(h\mathbf{x}_1, h\mathbf{x}_1)^{-1/2} \to |e(\mathbf{0})|k_e(\mathbf{0},\mathbf{0})^{-1/2}.$$

Using this bound in (25) then shows that the ratio on the left-hand side is bounded as $h \to 0$, since we have assumed $e(\mathbf{0}) \neq 0$. □

B.9 Proof of Proposition 12

Proof of Proposition 12. Recall that the estimator $\mathbf{r}_n^h[f]$ is any maximiser of

$$\mathcal{L}_n^h(\mathbf{r}) = -\underbrace{\left\{ f(X_n^h)^\top \mathbf{K}_{b_\mathbf{r},h}^{-1} f(X_n^h) - \frac{(\mathbf{1}^\top \mathbf{K}_{b_\mathbf{r},h}^{-1} f(X_n^h))^2}{\mathbf{1}^\top \mathbf{K}_{b_\mathbf{r},h}^{-1}\mathbf{1}} \right\}}_{=:Q_n^h(f,\mathbf{r})} - \log\det \mathbf{K}_{b_\mathbf{r},h}.$$

Maximising $\mathcal{L}_n^h(\mathbf{r})$ is equivalent to maximising

$$\mathcal{J}_n^h(\mathbf{r}) = \mathcal{L}_n^h(\mathbf{r}) - \mathcal{L}_n^h(\mathbf{r}_0).$$

Let $\bar{f}(\mathbf{x}) := f(\mathbf{x}) - f(\mathbf{0})$. By assumption, $\bar{f}$ is an element of the RKHS of the covariance function $k_{b_{\mathbf{r}_0}}$. According to Proposition 20, $Q_n^h(f, \mathbf{r}) = |m_n^h[f]|^2_{\mathcal{H}_k(\mathcal{X})} \geq 0$. Also from

Proposition 20 and the minimal norm characterisation of the interpolant, we obtain the h-independent bound $Q_n^h(f, \mathbf{r}_0) \leq \|\bar{f}\|^2_{\mathcal{H}_{k_{b_{\mathbf{r}_0}}}(\mathcal{X})}$. Therefore

$$\begin{aligned}\mathcal{J}_n^h(\mathbf{r}) &= Q_n^h(f, \mathbf{r}_0) + \log\det \mathbf{K}_{b_{\mathbf{r}_0},h} - Q_n^h(f, \mathbf{r}) - \log\det \mathbf{K}_{b_{\mathbf{r}},h} \\ &\leq \log\det \mathbf{K}_{b_{\mathbf{r}_0},h} - \log\det \mathbf{K}_{b_{\mathbf{r}},h} + \|\bar{f}\|^2_{\mathcal{H}_{k_{b_{\mathbf{r}_0}}}(\mathcal{X})}.\end{aligned}$$

Because the covariance matrix factorises as $\mathbf{K}_{b_{\mathbf{r}},h} = \mathbf{B}_{\mathbf{r},h}\mathbf{K}_{e,h}\mathbf{B}_{\mathbf{r},h}$, where $\mathbf{K}_{e,h}$ is the covariance matrix for k_e at X_n^h and $\mathbf{B}_{\mathbf{r},h}$ is a diagonal matrix with entries $b_{\mathbf{r}}(h\mathbf{x}_i)$, we get

$$\mathcal{J}_n^h(\mathbf{r}) \leq 2\sum_{i=1}^n \log \frac{b_{\mathbf{r}_0}(h\mathbf{x}_i)}{b_{\mathbf{r}}(h\mathbf{x}_i)} + \|\bar{f}\|^2_{\mathcal{H}_{k_{b_{\mathbf{r}_0}}}(\mathcal{X})}.$$

Then, since the class of error bounds $b_{\mathbf{r}}$ is monotonically parametrised, $\mathcal{J}_n^h(\mathbf{r}) \to -\infty$ as $h \to 0$ uniformly over $\mathbf{r} \in [\mathbf{0}, \mathbf{r}_0 - \boldsymbol{\epsilon}]$ for any $\boldsymbol{\epsilon} > \mathbf{0}$. Because $\mathcal{J}_n^h(\mathbf{r}_0) = 0$, this establishes that $\liminf_{h\to 0} \mathbf{r}_n^h[f] \geq \mathbf{r}_0$, as claimed. □

B.10 Calculations for Multidimensional Output

To simplify the presentation we assume throughout that $\sigma = 1$, since σ enters only as a multiplicative constant that can be propagated through the calculations at the end. Let $k_{\mathcal{X}}(\mathbf{x}, \mathbf{x}') := k_0^2 + b(\mathbf{x})b(\mathbf{x}')k_e(\mathbf{x}, \mathbf{x}')$. From the Kronecker decomposition $\mathbf{K} = \mathbf{K}_{\mathcal{X}} \otimes \mathbf{K}_{\mathcal{T}}$ we have that

$$\mathbf{K}^{-1} = (\mathbf{K}_{\mathcal{X}}^{-1}) \otimes (\mathbf{K}_{\mathcal{T}}^{-1})$$

where from (16) we know that

$$\mathbf{K}_{\mathcal{X}}^{-1} = \mathbf{K}_b^{-1} - \mathbf{K}_b^{-1}\mathbf{1}(k_0^{-2} + \mathbf{1}^\top\mathbf{K}_b^{-1}\mathbf{1})^{-1}\mathbf{1}^\top\mathbf{K}_b^{-1}.$$

Let $\mathbf{k}(\mathbf{x}, \mathbf{t})$ be the column vector with entries $k((\mathbf{x}_i, \mathbf{t}_i), (\mathbf{x}, \mathbf{t}))$, and analogously define $\mathbf{k}_{\mathcal{X}}(\mathbf{x})$ and $\mathbf{k}_{\mathcal{T}}(\mathbf{t})$ as the column vectors with respective entries $k_{\mathcal{X}}(\mathbf{x}_i, \mathbf{x})$ and $k_{\mathcal{T}}(\mathbf{t}_i, \mathbf{t})$. In this notation we have also the Kronecker decomposition $\mathbf{k}(\mathbf{x}, \mathbf{t}) = \mathbf{k}_{\mathcal{X}}(\mathbf{x}) \otimes \mathbf{k}_{\mathcal{T}}(\mathbf{t})$ and the algebraic result that $\mathbf{k}(\mathbf{x}, \mathbf{t})^\top = (\mathbf{k}_{\mathcal{X}}(\mathbf{x})^\top) \otimes (\mathbf{k}_{\mathcal{T}}(\mathbf{t})^\top)$. For the conditional mean function we therefore have that

$$\begin{aligned}m_n[f](\mathbf{x}, \mathbf{t}) &= \mathbf{k}(\mathbf{x}, \mathbf{t})^\top\mathbf{K}^{-1}f(X_n) \\ &= [(\mathbf{k}_{\mathcal{X}}(\mathbf{x})^\top) \otimes (\mathbf{k}_{\mathcal{T}}(\mathbf{t}))^\top][(\mathbf{K}_{\mathcal{X}}^{-1}) \otimes (\mathbf{K}_{\mathcal{T}}^{-1})]f(X_n) \\ &= [\mathbf{k}_{\mathcal{X}}(\mathbf{x})^\top\mathbf{K}_{\mathcal{X}}^{-1}] \otimes [\mathbf{k}_{\mathcal{T}}(\mathbf{t})^\top\mathbf{K}_{\mathcal{T}}^{-1}]f(X_n)\end{aligned}$$

where in the final equality we have exploited the *mixed-product* property of the Kronecker product. The dependence on k_0^2 of this expression occurs only in the term $\mathbf{k}_{\mathcal{X}}(\mathbf{x})^\top\mathbf{K}_{\mathcal{X}}^{-1}$. From the calculations in Appendix B.2, we can take the $k_0^2 \to \infty$ limit of the term $\mathbf{k}_{\mathcal{X}}(\mathbf{x})^\top\mathbf{K}_{\mathcal{X}}^{-1}$, to obtain that

$$m_n[f](\mathbf{x}, \mathbf{t}) = \left\{\mathbf{k}_b(\mathbf{x})^\top\mathbf{K}_b^{-1} + [1 - \mathbf{k}_b(\mathbf{x})^\top\mathbf{K}_b^{-1}\mathbf{1}]\frac{\mathbf{1}^\top\mathbf{K}_b^{-1}}{\mathbf{1}^\top\mathbf{K}_b^{-1}\mathbf{1}}\right\} \otimes \left[k_{\mathcal{T}}(\mathbf{t})^\top\mathbf{K}_{\mathcal{T}}^{-1}\right] f(X_n).$$

For the conditional covariance function, we have from a similar argument based on the mixed-product property that

$$\begin{aligned} k_n[f]((\mathbf{x},\mathbf{t}),(\mathbf{x}',\mathbf{t}')) &= k((\mathbf{x},\mathbf{t}),(\mathbf{x}',\mathbf{t}')) - \mathbf{k}(\mathbf{x},\mathbf{t})^\top \mathbf{K}^{-1}\mathbf{k}(\mathbf{x}',\mathbf{t}') \\ &= k_\mathcal{X}(\mathbf{x},\mathbf{x}')k_\mathcal{T}(\mathbf{t},\mathbf{t}') - \underbrace{[\mathbf{k}_\mathcal{X}(\mathbf{x})^\top \mathbf{K}_\mathcal{X}^{-1}\mathbf{k}_\mathcal{X}(\mathbf{x}')]}_{(*)}[\mathbf{k}_\mathcal{T}(\mathbf{t})^\top \mathbf{K}_\mathcal{T}^{-1}\mathbf{k}_\mathcal{T}(\mathbf{t}')]. \end{aligned} \tag{26}$$

The term $(*)$ can be read off from (15) and (17):

$$(*) = (k_0^2\mathbf{1} + \mathbf{k}_b(\mathbf{x}))^\top \left\{ \mathbf{K}_b^{-1} - \frac{\mathbf{K}_b^{-1}\mathbf{1}\mathbf{1}^\top \mathbf{K}_b^{-1}}{k_0^{-2} + \mathbf{1}^\top \mathbf{K}_b^{-1}\mathbf{1}} \right\} (k_0^2\mathbf{1} + \mathbf{k}_b(\mathbf{x}'))$$

The fractional term can again be treated using the Taylor expansion (18) for k_0^{-2} at 0, which upon expanding brackets yields

$$(*) = k_0^2 + \underbrace{\mathbf{k}_b(\mathbf{x})^\top \mathbf{K}_b^{-1}\mathbf{k}_b(\mathbf{x}') - \frac{[\mathbf{k}_b(\mathbf{x})^\top \mathbf{K}_b^{-1}\mathbf{1} - 1][\mathbf{k}_b(\mathbf{x}')^\top \mathbf{K}_b^{-1}\mathbf{1} - 1]^\top}{\mathbf{1}^\top \mathbf{K}_b^{-1}\mathbf{1}} + O(k_0^{-2})}_{(**)}.$$

Substituting this expression into (26), and using the definition of $k_\mathcal{X}$, we obtain that

$$\begin{aligned} k_n[f]((\mathbf{x},\mathbf{t}),(\mathbf{x}',\mathbf{t}')) &= (k_0^2 + k_b(\mathbf{x},\mathbf{x}'))k_\mathcal{T}(\mathbf{t},\mathbf{t}') - (k_0^2 + (**))[\mathbf{k}_\mathcal{T}(\mathbf{t})^\top \mathbf{K}_\mathcal{T}^{-1}\mathbf{k}_\mathcal{T}(\mathbf{t}')] \\ &= k_0^2 \underbrace{[k_\mathcal{T}(\mathbf{t},\mathbf{t}') - \mathbf{k}_\mathcal{T}(\mathbf{t})^\top \mathbf{K}_\mathcal{T}^{-1}\mathbf{k}_\mathcal{T}(\mathbf{t}')]}_{(***)} \\ &\quad + k_b(\mathbf{x},\mathbf{x}')k_\mathcal{T}(\mathbf{t},\mathbf{t}') - (**)[\mathbf{k}_\mathcal{T}(\mathbf{t})^\top \mathbf{K}_\mathcal{T}^{-1}\mathbf{k}_\mathcal{T}(\mathbf{t}')] \end{aligned}$$

where for $\mathbf{t},\mathbf{t}'$ in the training set we have $(***) = 0$, which gives the desired result.

C Details for the Cardiac Model

This appendix summarises the most important aspects of the cardiac model that we used, and is principally intended for researchers working in the area of cardiac modelling who are interested in understanding the technical modelling aspects of the case study that we report in Section 3.

Geometry The model was based on a heart geometry obtained from a computer tomography (CT) dataset of a single patient, used in Strocchi et al. [2020, 2023]. The original patient geometry was recorded using an average mesh resolution of 1.06 ± 0.16 mm; below we describe how our coarser and finer meshes were constructed. Fibres in the atria and the ventricles were generated with *universal atrial coordinates* [Roney et al., 2019] and a rule-based method developed by Bayer et al. [2012]. Full details about the geometry are provided in Strocchi et al. [2020, 2023].

Atrial and Ventricular Activation For the cardiac dynamics, we simulated atrial and ventricular activation using an *Eikonal model* [Neic et al., 2017]

$$\begin{aligned} \sqrt{\nabla t_a(\mathbf{x})^\top \mathbf{V}(\mathbf{x}) \nabla t_a(\mathbf{x})} &= 1 \qquad \mathbf{x} \in \Omega \\ t_a(\mathbf{x}) &= t_0 \qquad \mathbf{x} \in \Gamma \end{aligned}$$

where $t_a(\mathbf{x})$ is the local activation time in the active domain Ω, $\mathbf{V}$ is the tensor of the squared conduction velocities (CV) in the fibres, sheet and normal directions, and Γ is a subset of nodes in the domain that get activated at time t_0.

Myocardium The cardiac model that we used treats the myocardium as a transversely isotropic conduction medium. The fibre CV in the atrial and ventricular myocardium was set to 0.6 m/s and 0.9 m/s, consistent with experimental measurements [Taggart et al., 2000, Hansson et al., 1998], while the anisotropy ratio was set to 0.4 in all tissues [Strocchi et al., 2023]. We simulated fast endocardial conduction in the ventricles by defining a 1 mm thick layer and assigning it with increased CV by five fold compared to normal ventricular myocardium [Ono et al., 2009]. Fast conduction in the Bachmann bundle area in the atria was simulated by defining a region between the left and the right atrium as in Strocchi et al. [2023], and by assigning it with CV increased by 3.5 fold compared to normal atrial myocardium [Strocchi et al., 2023]. The atria and ventricles were electrically isolated by defining an insulating layer to avoid non-physiological activation and to control the atrio-ventricular delay. Atrial and ventricular activation were initiated at the site of the right atrial and right ventricular pacing lead, respectively, identified from the CT images. The atrio-ventricular delay was set to 150 ms.

Active Tension Atrial and ventricular activation triggered a rise in local transmembrane potential [Neic et al., 2017], which then triggered a rise in active tension. Active tension was modelled with a phenomenological model developed by Niederer et al. [2011]:

$$\begin{aligned} S_a(\mathbf{x}, t) &= T_{\text{peak}} \phi(\lambda) \tanh^2 \left(\frac{t_s}{\tau_r} \right) \tanh^2 \left(\frac{t_{\text{dur}} - t_s}{\tau_d} \right), \qquad 0 < t_s < t_{\text{dur}} \ , \\ \phi(\lambda) &= \tan(\text{ld}(\lambda - \lambda_0)) \ , \qquad t_s = t - t_{\text{a}}(\mathbf{x}) - t_{\text{emd}} \end{aligned}$$

where T_{peak}, τ_r, τ_d, t_{dur}, ld, λ_0 and t_{emd} represent the reference tension, the rise and the decay time, the twitch duration, the reference stretch and the electro-mechanical delay. The following parameter values were used:

	T_{peak}<br>kPa	τ_r<br>ms	τ_d<br>ms	t_{dur}<br>ms	ld<br>-	λ_0<br>-	t_{emd}<br>ms
**Atria**	80	100	50	450	0.7	6	20
**Ventricles**	60	50	50	200	0.7	6	20

Passive Properties We simulated passive properties of atrial and ventricular myocardium with a transversely isotropic Guccione law

$$\Psi(\mathbf{E}) = \frac{C}{2}\left[e^{Q} - 1\right] ,$$
$$Q = b_{\mathrm{ff}} E_{\mathrm{ff}}^2 + 2 b_{\mathrm{fs}} (E_{\mathrm{fs}}^2 + E_{\mathrm{fn}}^2) + b_{\mathrm{ss}} (E_{\mathrm{ss}}^2 + E_{\mathrm{nn}}^2 + 2 E_{\mathrm{sn}}^2) ,$$

where f, s, n in the Cauchy–Green strain tensor $\mathbf{E}$ represent the strain in the local fibres, sheet and normal to sheet directions, and C, b_{ff}, b_{fs} b_{ss} are the bulk stiffness, and the stiffness in the fibre, cross-fibre and transverse directions. The following parameter values were used:

	C<br>kPa	b_{ff}<br>-	b_{fs}<br>-	b_{ss}<br>-
**Atria**	3	25	11	9
**Ventricles**	4	25	11	9

Parameters in tissues other than the atria and the ventricles were represented with a neo-Hookean law and the parameters were set according to Strocchi et al. [2020, 2023]. For all tissues, near-incompressibility was enforced with a penalty method with a bulk modulus $\kappa = 1000.0$ kPa. To constrain the motion of the heart, we applied omni-directional springs on the superior vena cava and the two right pulmonary veins, as well as a region around the apex (Figure 5, left). Spring stiffness was set to 0.1 kPa/μm.

Adjusting the Spatial Resolution We used `meshtool`, open-source software for mesh manipulation [Neic et al., 2020], to refine or coarsen the mesh to a desired spatial resolution for the experiments that we report. The spatial resolution x_1 that we used for extrapolation in the main text is the *nominal* resolution fed into `meshtool`, but we note that the nominal resolution is typically not exactly achieved in the re-meshing process; we therefore also report the effective average mesh resolution:

**Target dx** [mm]	**Effective dx** [mm]	**# nodes**	**# elements**
1.7	1.7	119,104	540,621
1.4	1.36	213,580	1,018,699
1.0	1.06	417,863	1,988,945
0.7	0.86	764,094	3,679,517
0.6	0.7	1,498,007	7,512,728
0.5	0.56	3,006,080	15,768,118
0.4	0.43	6,217,838	30,699,422

Computational Resources All simulations were carried out using the *Cardiac Arrhythmia Research Package* (CARP) [Vigmond et al., 2003, Augustin et al., 2021] on ARCHER2, a UK national super computing service (`https://www.archer2.ac.uk/`).

References

C. P. Arroyo, J. Dombard, F. Duchaine, L. Gicquel, B. Martin, N. Odier, and G. Staffelbach. Towards the large-eddy simulation of a full engine: Integration of a 360 azimuthal degrees fan, compressor and combustion chamber. Part I: Methodology and initialisation. *Journal of the Global Power and Propulsion Society*, page 133115, 2021.

C. M. Augustin, M. A. Gsell, E. Karabelas, E. Willemen, F. W. Prinzen, J. Lumens, E. J. Vigmond, and G. Plank. A computationally efficient physiologically comprehensive 3D–0D closed-loop model of the heart and circulation. *Computer Methods in Applied Mechanics and Engineering*, 386:114092, 2021.

F. Bach. On the effectiveness of Richardson extrapolation in data science. *SIAM Journal on Mathematics of Data Science*, 3(4):1251–1277, 2021.

J. D. Bayer, R. C. Blake, G. Plank, and N. A. Trayanova. A novel rule-based algorithm for assigning myocardial fiber orientation to computational heart models. *Annals of Biomedical Engineering*, 40:2243–2254, 2012.

J. Bect, S. Zio, G. Perrin, C. Cannamela, and E. Vazquez. On the quantification of discretization uncertainty: Comparison of two paradigms. In *14th World Congress in Computational Mechanics and ECCOMAS Congress 2020 (WCCM-ECCOMAS)*, 2021.

A. Berlinet and C. Thomas-Agnan. *Reproducing Kernel Hilbert Spaces in Probability and Statistics*. Springer Science & Business Media, 2011.

C. A. Beschle and A. Barth. Quasi continuous level Monte Carlo for random elliptic PDEs. In *International Conference on Monte Carlo and Quasi-Monte Carlo Methods in Scientific Computing*, pages 3–31. Springer, 2022.

C. Brezinski. Composite sequence transformations. *Numerische Mathematik*, 46:311–321, 1985.

C. Brezinski. A survey of iterative extrapolation by the E-algorithm, Det Kong. *Det Kongelige Norske Videnskabers Selskabs Skrifter*, 2:1–26, 1989.

C. Brezinski and M. R. Zaglia. *Extrapolation Methods: Theory and Practice*. Elsevier, 2013.

R. Bulirsch and J. Stoer. Fehlerabschätzungen und Extrapolation mit rationalen Funktionen bei Verfahren vom Richardson-Typus. *Numerische Mathematik*, 6(1):413–427, 1964.

V. Cabannes and S. Vigogna. How many samples are needed to leverage smoothness? In *Proceedings of the 38th Conference on Neural Information Processing Systems*, 2024.

L. Chizat, P. Roussillon, F. Léger, F.-X. Vialard, and G. Peyré. Faster Wasserstein distance estimation with the Sinkhorn divergence. In *Proceedings of the 34th Conference on Neural Information Processing Systems*, 2020.

J. Cockayne, C. J. Oates, T. J. Sullivan, and M. Girolami. Bayesian probabilistic numerical methods. *SIAM Review*, 61(4):756–789, 2019.

P. S. Craig, M. Goldstein, A. Seheult, and J. Smith. Constructing partial prior specifications for models of complex physical systems. *Journal of the Royal Statistical Society, Series D*, 47(1):37–53, 1998.

J. A. Cumming and M. Goldstein. Small sample Bayesian designs for complex high-dimensional models based on information gained using fast approximations. *Technometrics*, 51(4):377–388, 2009.

J.-P. Delahaye. Automatic selection of sequence transformations. *Mathematics of Computation*, 37(155):197–204, 1981.

A. Durmus, U. Simsekli, E. Moulines, R. Badeau, and G. Richard. Stochastic gradient Richardson-Romberg Markov chain Monte Carlo. In *Proceedings of the 30th Conference on Neural Information Processing Systems*, 2016.

A. Ehara and S. Guillas. An adaptive strategy for sequential designs of multilevel computer experiments. *International Journal for Uncertainty Quantification*, 13(4), 2023.

B. Germain-Bonne. Convergence acceleration of number-machine sequences. *Journal of Computational and Applied Mathematics*, 32(1-2):83–88, 1990.

J. Han, A. Jentzen, and W. E. Solving high-dimensional partial differential equations using deep learning. *Proceedings of the National Academy of Sciences*, 115(34):8505–8510, 2018.

A. Hansson, M. Holm, P. Blomström, R. Johansson, C. Lührs, J. Brandt, and S. Olsson. Right atrial free wall conduction velocity and degree of anisotropy in patients with stable sinus rhythm studied during open heart surgery. *European Heart Journal*, 19(2):293–300, 1998.

I. M. Held. The gap between simulation and understanding in climate modeling. *Bulletin of the American Meteorological Society*, 86(11):1609–1614, 2005.

P. Hennig, M. A. Osborne, and M. Girolami. Probabilistic numerics and uncertainty in computations. *Proceedings of the Royal Society A: Mathematical, Physical and Engineering Sciences*, 471(2179):20150142, 2015.

P. E. Jacob, J. O'Leary, and Y. F. Atchadé. Unbiased Markov chain Monte Carlo methods with couplings. *Journal of the Royal Statistical Society, Series B*, 82(3):543–600, 2020.

Y. Ji, H. S. Yuchi, D. Soeder, J.-F. Paquet, S. A. Bass, V. R. Joseph, C. J. Wu, and S. Mak. Conglomerate multi-fidelity Gaussian process modeling, with application to heavy-ion collisions. *SIAM/ASA Journal on Uncertainty Quantification*, 12(2):473–502, 2024.

T. Karvonen. Small sample spaces for Gaussian processes. *Bernoulli*, 29(2):875–900, 2023.

T. Karvonen, C. J. Oates, and S. Särkkä. A Bayes–Sard cubature method. In *Proceedings of the 32nd Conference on Neural Information Processing Systems*, 2018.

T. Karvonen, G. Wynne, F. Tronarp, C. Oates, and S. Sarkka. Maximum likelihood estimation and uncertainty quantification for Gaussian process approximation of deterministic functions. *SIAM/ASA Journal on Uncertainty Quantification*, 8(3):926–958, 2020.

M. C. Kennedy and A. O'Hagan. Predicting the output from a complex computer code when fast approximations are available. *Biometrika*, 87(1):1–13, 2000.

F. Larkin. Some techniques for rational interpolation. *The Computer Journal*, 10(2):178–187, 1967.

M. Ledoux and M. Talagrand. *Probability in Banach Spaces: Isoperimetry and Processes.* Springer Science & Business Media, 1991.

V. Lemaire and G. Pagès. Multilevel Richardson–Romberg extrapolation. *Bernoulli*, 20(3): 1029–1067, 2017.

D. J. Lucia, P. S. Beran, and W. A. Silva. Reduced-order modeling: New approaches for computational physics. *Progress in Aerospace Sciences*, 40(1-2):51–117, 2004.

W. Madych and S. Nelson. Bounds on multivariate polynomials and exponential error estimates for multiquadric interpolation. *Journal of Approximation Theory*, 70(1):94–114, 1992.

A. J. Majda and B. Gershgorin. Quantifying uncertainty in climate change science through empirical information theory. *Proceedings of the National Academy of Sciences*, 107(34): 14958–14963, 2010.

H. Mhaskar, F. Narcowich, and J. Ward. Spherical Marcinkiewicz-Zygmund inequalities and positive quadrature. *Mathematics of Computation*, 70(235):1113–1130, 2001.

A. Neic, F. O. Campos, A. J. Prassl, S. A. Niederer, M. J. Bishop, E. J. Vigmond, and G. Plank. Efficient computation of electrograms and ECGs in human whole heart simulations using a reaction-eikonal model. *Journal of Computational Physics*, 346:191–211, 2017.

A. Neic, M. A. Gsell, E. Karabelas, A. J. Prassl, and G. Plank. Automating image-based mesh generation and manipulation tasks in cardiac modeling workflows using meshtool. *SoftwareX*, 11:100454, 2020.

S. A. Niederer, G. Plank, P. Chinchapatnam, M. Ginks, P. Lamata, K. S. Rhode, C. A. Rinaldi, R. Razavi, and N. P. Smith. Length-dependent tension in the failing heart and the efficacy of cardiac resynchronization therapy. *Cardiovascular Research*, 89(2):336–343, 2011.

N. Ono, T. Yamaguchi, H. Ishikawa, M. Arakawa, N. Takahashi, T. Saikawa, and T. Shimada. Morphological varieties of the purkinje fiber network in mammalian hearts, as revealed by light and electron microscopy. *Archives of Histology and Cytology*, 72(3):139–149, 2009.

V. I. Paulsen and M. Raghupathi. *An Introduction to the Theory of Reproducing Kernel Hilbert Spaces*. Cambridge University Press, 2016.

B. Peherstorfer, K. Willcox, and M. Gunzburger. Survey of multifidelity methods in uncertainty propagation, inference, and optimization. *SIAM Review*, 60(3):550–591, 2018.

P. Piperni, A. DeBlois, and R. Henderson. Development of a multilevel multidisciplinary-optimization capability for an industrial environment. *AIAA Journal*, 51(10):2335–2352, 2013.

E. Porcu, M. Bevilacqua, R. Schaback, and C. J. Oates. The Matérn model: A journey through statistics, numerical analysis and machine learning. *Statistical Science*, 2024. To appear.

C. E. Rasmussen and C. K. Williams. *Gaussian Processes for Machine Learning*. Springer, 2006.

C.-H. Rhee and P. W. Glynn. Unbiased estimation with square root convergence for SDE models. *Operations Research*, 63(5):1026–1043, 2015.

L. F. Richardson. The approximate arithmetical solution by finite differences of physical problems involving differential equations, with an application to the stresses in a masonry dam. *Philosophical Transactions of the Royal Society A*, 210(459-470):307–357, 1911.

L. F. Richardson and J. A. Gaunt. The deferred approach to the limit. *Philosophical Transactions of the Royal Society A*, 226:223–361, 1927.

C. H. Roney, A. Pashaei, M. Meo, R. Dubois, P. M. Boyle, N. A. Trayanova, H. Cochet, S. A. Niederer, and E. J. Vigmond. Universal atrial coordinates applied to visualisation, registration and construction of patient specific meshes. *Medical Image Analysis*, 55:65–75, 2019.

J. Sacks, W. J. Welch, T. J. Mitchell, and H. P. Wynn. Design and analysis of computer experiments. *Statistical Science*, 4(4):409–423, 1989.

D. Shanks. Non-linear transformations of divergent and slowly convergent sequences. *Journal of Mathematics and Physics*, 34(1-4):1–42, 1955.

A. Sidi. *Practical Extrapolation Methods: Theory and Applications*. Cambridge University Press, 2003.

M. L. Stein. *Interpolation of Spatial Data: Some Theory for Kriging*. Springer Science & Business Media, 1999.

M. L. Stein and Y. Hung. Comment on "Probabilistic integration: A role in statistical computation?". *Statistical Science*, 34(1):34–37, 2019.

M. Strocchi, M. A. Gsell, C. M. Augustin, O. Razeghi, C. H. Roney, A. J. Prassl, E. J. Vigmond, J. M. Behar, J. S. Gould, C. A. Rinaldi, et al. Simulating ventricular systolic motion in a four-chamber heart model with spatially varying robin boundary conditions to model the effect of the pericardium. *Journal of Biomechanics*, 101:109645, 2020.

M. Strocchi, S. Longobardi, C. M. Augustin, M. A. Gsell, A. Petras, C. A. Rinaldi, E. J. Vigmond, G. Plank, C. J. Oates, R. D. Wilkinson, et al. Cell to whole organ global sensitivity analysis on a four-chamber heart electromechanics model using Gaussian processes emulators. *PLOS Computational Biology*, 19(6):e1011257, 2023.

P. Taggart, P. M. Sutton, T. Opthof, R. Coronel, R. Trimlett, W. Pugsley, and P. Kallis. Inhomogeneous transmural conduction during early ischaemia in patients with coronary artery disease. *Journal of Molecular and Cellular Cardiology*, 32(4):621–630, 2000.

O. Teymur, C. Foley, P. Breen, T. Karvonen, and C. J. Oates. Black box probabilistic numerics. In *Proceedings of the 35th Conference on Neural Information Processing Systems*, 2021.

T. N. Thiele. *Interpolationsrechnung*. BG Teubner, 1909.

P. Thodoroff, M. Kaiser, R. Williams, R. Arthern, S. Hosking, N. Lawrence, J. Byrne, and I. Kazlauskaite. Multi-fidelity experimental design for ice-sheet simulation. *arXiv preprint arXiv:2307.08449*, 2023.

R. Tuo, C. J. Wu, and D. Yu. Surrogate modeling of computer experiments with different mesh densities. *Technometrics*, 56(3):372–380, 2014.

E. J. Vigmond, M. Hughes, G. Plank, and L. J. Leon. Computational tools for modeling electrical activity in cardiac tissue. *Journal of Electrocardiology*, 36:69–74, 2003.

H. Wendland. *Scattered Data Approximation*. Cambridge University Press, 2004.
